# Machine Learning-Based Analysis of Optical Coherence Tomography Angiography Images for Age-Related Macular Degeneration

**DOI:** 10.3390/biomedicines13092152

**Published:** 2025-09-05

**Authors:** Abdullah Alfahaid, Tim Morris, Tim Cootes, Pearse A. Keane, Hagar Khalid, Nikolas Pontikos, Fatemah Alharbi, Easa Alalwany, Abdulqader M. Almars, Amjad Aldweesh, Abdullah G. M. ALMansour, Panagiotis I. Sergouniotis, Konstantinos Balaskas

**Affiliations:** 1 Department of Computer Science, College of Computer Science and Engineering at Yanbu, Taibah University, Medina 46421, Saudi Arabia; fmhharbi@taibahu.edu.sa (F.A.); ealwani@taibahu.edu.sa (E.A.); amars@taibahu.edu.sa (A.M.A.); 2 Moorfields Eye Hospital, 162 City Road, London EC1V 2PD, UKhagar.khalid@nhs.net (H.K.);; 3 School of Computer Science, The University of Manchester, Oxford Road, Manchester M13 9PL, UK; 4 Centre for Imaging Sciences, The University of Manchester, Oxford Road, Manchester M13 9PL, UK; timothy.f.cootes@manchester.ac.uk; 5 Institute of Ophthalmology, University College London, 11-43 Bath Street, London EC1V 9EL, UK; 6 College of Computing and Information Technology, Shaqra University, Shaqra 11911, Saudi Arabia; a.aldweesh@su.edu.sa; 7 Radiology and Medical Imaging Department, College of Applied Medical Sciences, Prince Sattam Bin Abdulaziz University, Alkharj 11942, Saudi Arabia; ag.alqahtani@psau.edu.sa; 8 School of Biological Sciences, The University of Manchester, Oxford Road, Manchester M13 9PL, UK; 9 Manchester Royal Eye Hospital, Oxford Road, Manchester M13 9WL, UK

**Keywords:** age-related macular degeneration (AMD), choroidal neovascularisation (CNV), optical coherence tomography angiography (OCTA), texture analysis, automated diagnosis

## Abstract

**Background/Objectives:** Age-related macular degeneration (AMD) is the leading cause of visual impairment among the elderly. Optical coherence tomography angiography (OCTA) is a non-invasive imaging modality that enables detailed visualisation of retinal vascular layers. However, clinical assessment of OCTA images is often challenging due to high data volume, pattern variability, and subtle abnormalities. This study aimed to develop automated algorithms to detect and quantify AMD in OCTA images, thereby reducing ophthalmologists’ workload and enhancing diagnostic accuracy. **Methods:** Two texture-based algorithms were developed to classify OCTA images without relying on segmentation. The first algorithm used whole local texture features, while the second applied principal component analysis (PCA) to decorrelate and reduce texture features. Local texture descriptors, including rotation-invariant uniform local binary patterns ($LBP^{2riu}$), local binary patterns (LBP), and binary robust independent elementary features (BRIEF), were combined with machine learning classifiers such as support vector machine (SVM) and K-nearest neighbour (KNN). OCTA datasets from Manchester Royal Eye Hospital and Moorfields Eye Hospital, covering healthy, dry AMD, and wet AMD eyes, were used for evaluation. **Results:** The first algorithm achieved a mean area under the receiver operating characteristic curve (AUC) of 1.00±0.00 for distinguishing healthy eyes from wet AMD. The second algorithm showed superior performance in differentiating dry AMD from wet AMD (AUC 0.85±0.02). **Conclusions:** The proposed algorithms demonstrate strong potential for rapid and accurate AMD diagnosis in OCTA workflows. By reducing manual image evaluation and associated variability, they may support improved clinical decision-making and patient care.

## 1. Introduction

Age-related macular degeneration (AMD) is a very complex, heterogeneous retinal disorder that is a common cause of vision damage in the elderly population [1,2]. AMD primarily affects the macula, the central region of the retina. It is clinically divided into two different types, namely wet AMD and dry AMD [3]. Wet AMD is characterised by the presence of choroidal neovascularisation (CNV), which involves the growth of abnormal blood vessels and the presence of fluid in the central retina [3]. Dry AMD involves outer retinal thinning and is characterised by degeneration of retinal pigment epithelial cells, and underlying choroidal capillaries [3]. Dry AMD is the more common subtype and is linked to gradual vision loss, while wet AMD is associated with more rapid vision impairment [3]. Notably, wet AMD can be successfully treated with intravitreal injections. As a result, early discovery and treatment are critical, and quick diagnosis has been associated with better results [4]. Early detection of areas related to CNV lesions and the distinction between subjects with wet AMD and dry AMD are consequently prioritised in terms of effort and healthcare resources.

To aid the assessment and management of AMD, various retinal vasculature imaging modalities have been developed. Optical coherence tomography angiography (OCTA) is a dye-free imaging technology that involves non-invasive volumetric three-dimensional imaging. OCTA provides detailed visualisation of blood circulation in the retinal vascular layers and, in contrast to other recognised fundus imaging techniques, such as fluorescein angiography (FA) and indocyanine green angiography (IGA), OCTA is both rapid and non-invasive [5,6,7]. OCTA characterises both moving and static elements in the retinal and choroidal vessels, enabling the visualisation of vascular abnormalities and other vascular details that can assist in differentiating healthy vascular appearance from that associated with dry and wet AMD.

**Figure 1:**
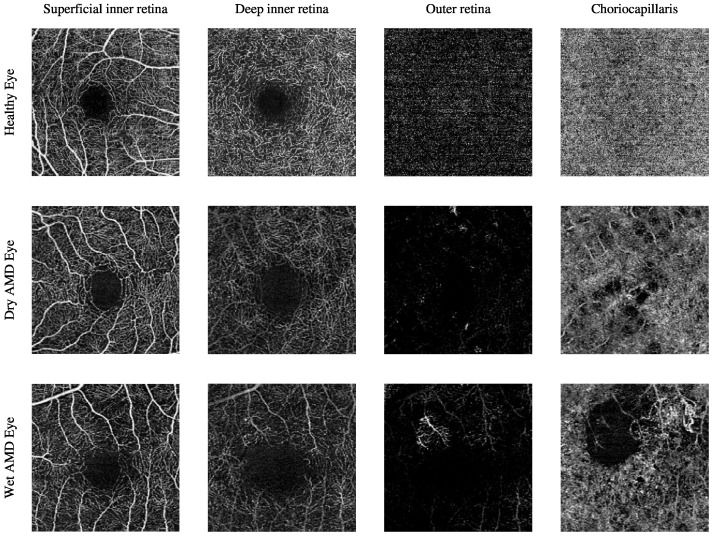
The textural appearance of the blood vessels network in the superficial inner retina, deep inner retina, outer retina, and choriocapillaris layers in OCTA images, captured from a 3 mm × 3 mm field of view centred around the fovea. Each row illustrates a different eye condition from the various ocular vascular layers. The first row shows a healthy eye, the second row shows a dry AMD eye, and the final row shows a wet AMD eye.

The current clinical practice for detecting CNV lesions and assessing treatment effectiveness in wet AMD involves visually evaluating the textural appearance of OCTA images [8,9]. However, this process remains challenging due to the substantial volume of image data per OCTA scan, individual variations in textural patterns, and the visual similarity of CNV, non-CNV, and healthy vascular regions across different patients [10]. The textural appearance of retinal vascular layers in OCTA images for eyes with varying conditions is illustrated in Figure 1. These images demonstrate vascular layers in eyes without vascular pathologies, with dry AMD, and with wet AMD, underscoring the intricate patterns of blood vessels across the different layers.

OCTA has become an essential modality for diagnosing and monitoring AMD, particularly for detecting and characterising CNV in wet AMD. Its depth-resolved imaging and ability to visualise microvascular flow without dye injection allow for more precise delineation of neovascular membranes. Specifically, OCTA facilitates direct visualisation and measurement of CNV area, location, vessel density, and flow patterns [7,11]. Unlike FA and IGA, which often obscure neovascular details due to dye leakage, OCTA offers high-resolution, dye-free images that preserve vascular integrity. This feature enables a more accurate assessment of disease progression and the effectiveness of anti-VEGF treatments in clinical follow-up [12,13].

OCTA imaging enables the visualisation of the vascular architecture within four depth-resolved layers: the superficial inner retina, the deep inner retinal layer, the outer retinal layer, and the choriocapillaris. Each layer contributes specific insights into AMD pathology. The superficial and deep inner retinal layers, although more commonly affected in diabetic retinopathy and glaucoma, may also show changes in AMD patients. These include reduced capillary density and flow voids, potentially linked to retinal pigment epithelium (RPE) dysfunction or secondary ischaemic processes. The outer retinal layer may display hyperintense vascular networks in cases of neovascular AMD, indicating pathological invasion of new vessels from the choroid through Bruch’s membrane. The choriocapillaris layer is particularly relevant in AMD and, among others, OCTA can detect choriocapillaris dropout and hypoperfusion that may precede or accompany RPE degeneration in dry AMD [11,14].

These vascular changes, CNV branching patterns, capillary non-perfusion, and choriocapillaris signal attenuation can be quantitatively assessed using OCTA-derived metrics including vessel density, perfusion density, and CNV area. Such quantitative analyses are key for monitoring disease progression, evaluating therapeutic outcomes, and identifying patients at increased risk of progressing from dry to wet AMD [7,12]. By enabling non-invasive, high-resolution visualisation of these vascular alterations, OCTA provides clinicians with an invaluable tool for both diagnosis and prognosis.

The texture of images contains rich details describing complex visual patterns distinguishable by brightness, size, or shape [15]. In medical imaging, texture information relates to the macro- and micro-structural properties of images representing biomedical tissues [16]. Clinicians are trained to interpret, establish standardised qualitative features, and link visual texture patterns to specific pathologies in medical images. Early attempts to identify ocular vascular pathologies related to AMD in OCTA image data focused on qualitative analysis approaches [8,9,17,18,19,20,21,22].

However, qualitative features often fail to fully describe texture characteristics and are limited to low-level terms like uniformness, randomness, smoothness, and coarseness, whereas human perception of textures is far richer [16]. Differences in mapping patterns may lead to interpretation errors with undesirable consequences [16,23,24,25]. These issues stem from the complexity of human biology, anatomy, image acquisition techniques, and observer training [16], worsened by the absence of specific diagnostic criteria for OCTA images in retinal diseases like AMD [13]. Moreover, recognising textural information related to higher-order statistics or spectral properties is challenging for the human eye [26].

Consequently, automating OCTA image analysis is expected to assist ophthalmologists in extracting meaningful features that may be visually challenging to distinguish. Additional benefits include reducing ophthalmologists’ workload while enhancing efficiency, consistency, and reliability in clinical diagnosis. Automation can reduce patient waiting times and dependency on subjective interpretation.

To overcome the challenges identified in the literature, this study presents two automated methods for detecting and quantifying AMD in OCTA image data. The first algorithm is built upon the $LBP^{2riu}$ descriptor [27]. This descriptor extracts local texture features that are invariant to illumination changes and rotation, making it highly suitable for analysing subtle vascular texture patterns in OCTA images. The extracted $LBP^{2riu}$ features are directly used by a supervised classifier that distinguishes between healthy, dry AMD, and wet AMD images. The second algorithm extends the first by incorporating a dimensionality reduction step using PCA. The $LBP^{2riu}$ features are first extracted and then transformed into a lower-dimensional space using PCA, yielding a hybrid descriptor referred to as $LBP^{2riuPCA}$. This transformation captures the most significant variance in the texture patterns and suppresses noise and redundancy. The resulting features are subsequently used by a supervised classifier that distinguishes between healthy, dry AMD, and wet AMD images. These algorithmic pipelines are designed to be fully automated, with no requirement for manual feature selection or expert intervention, ensuring clinical scalability and reproducibility. In both cases, the trained classifiers were evaluated for three distinct diagnostic tasks: distinguishing healthy from wet AMD images, differentiating dry from wet AMD, and identifying CNV versus non-CNV lesions. The algorithms demonstrated high accuracy and robustness across OCTA image datasets obtained from two independent hospitals.

Therefore, the key contributions are summarised as follows:

- Development of two domain-specific texture descriptors for AMD detection in OCTA images: a supervised descriptor based on greyscale and rotation-invariant uniform local binary patterns ($LBP^{2riu}$) [27], and a hybrid descriptor ($LBP^{2riuPCA}$) that integrates $LBP^{2riu}$ with principal component analysis (PCA).
- Construction of two fully automated classification algorithms for AMD detection. The first algorithm utilises $LBP^{2riu}$ extracted features. While the second algorithm leverages $LBP^{2riuPCA}$ transformed features.
- The proposed techniques have contributed to the development of three diagnostic applications for OCTA-based AMD pathology detection: classifying healthy images from those with wet AMD, differentiating between dry and wet AMD images, and identifying OCTA images with CNV lesions versus non-CNV lesions.
- These algorithmic pipelines are evaluated using diverse OCTA image datasets from two hospitals, the Manchester Royal Eye Hospital and the Moorfields Eye Hospital, demonstrating promising results.

### Related Works

Several studies [8,9,18,19,20,21,22,28,29,30,31,32,33] have focused on quantifying and identifying AMD in OCTA images. However, automated detection of AMD in OCTA data has received limited attention. Recent advancements in automating OCTA texture analysis for AMD generally fall into two categories: image segmentation and image classification.

The automation of OCTA image texture analysis through image segmentation aims to partition the image into disjoint regions, simplifying its complex representation into a more interpretable form by highlighting key areas such as CNV regions. Recent studies addressing automated segmentation and quantification of CNV lesions in OCTA images for AMD patients include works by Jia et al. [6], Liu et al. [10], Zhang et al. [34], and Taibouni et al. [35].

Jia et al. [6] proposed an early automated segmentation approach for analysing OCTA images to detect and quantify CNV lesion regions. Their method utilises two-dimensional greyscale OCTA images of the deep inner and outer retinal layers, captured from a 3 mm × 3 mm field of view centred around the fovea, with imaging depth manually adjusted to highlight CNV areas. Involving 10 eyes (five with wet AMD and five normal controls), their segmentation begins by applying a 30×30-pixel Gaussian filter to the deep inner retinal layer image to create a binary map of large vessel projections, removing superficial blood flow artefacts. This filtered image is subtracted from the outer retinal layer image to eliminate large vessel projections, followed by a 10×10-pixel Gaussian filter to produce a binary outer retinal flow map devoid of residual projections. This process yields a clean map of the typically avascular outer retinal layer, enabling further analysis, such as measuring CNV area size.

The segmentation scheme proposed by Liu et al. [10], building upon the work of Jia et al. [6], introduces enhancements for accurate recognition of choroidal neovascularisation (CNV) areas in greyscale OCTA images of the deep inner and outer retinal layers, captured from a 3 mm × 3 mm field of view centred around the fovea. The study involved OCTA images manually adjusted for optimal imaging depth from seven eyes of participants diagnosed with wet AMD. Their method, which assumes the CNV region occupies a large portion of the OCTA image, begins with pre-processing using a 20×20-pixel Gaussian filter, followed by subtraction to highlight CNV regions by removing deep inner retinal blood vessels. A context-aware saliency model based on orientation, brightness, and location refines the CNV area by eliminating noise and generating a saliency map, which is further processed through nonlinear filtering, thresholding, and morphological operations to delineate the CNV boundary. The final CNV area is measured by estimating the proportion of flow pixels within the boundary [6,10].

Zhang et al. [34] proposed an automated segmentation algorithm for identifying CNV lesions and quantifying the size of CNV regions in OCTA images, using two-dimensional greyscale images of the outer retinal layers captured from 3 mm × 3 mm and 6 mm × 6 mm fields of view centred around the fovea. The study involved 27 eyes from 23 AMD-diagnosed subjects, employing a semi-automated segmentation procedure with manual corrections to imaging depth levels for precise visualisation of CNV lesions. The algorithm follows six steps: inputting an OCTA image, enhancing contrast via adaptive thresholding, smoothing with a Gaussian filter, thresholding to create a binary image, applying morphological dilation to detect CNV boundaries, and estimating lesion size based on pixel proportions. Results showed reliable CNV measurements with 3 mm × 3 mm OCTA images, but challenges in accurately quantifying CNV regions with 6 mm × 6 mm images.

Taibouni et al. [35] developed an automated quantification and segmentation algorithm to distinguish various shapes, sizes, and locations of CNV lesions in AMD patients using two-dimensional greyscale OCTA images of the outer retinal layers, captured from a 3 mm × 3 mm field of view centred on the macula. The study included 54 eyes from 54 wet AMD-diagnosed patients, with manual adjustments to imaging depth levels ensuring optimal visualisation of CNV regions. Patients were divided into two categories based on CNV lesion topology: densely crowded networks without branching patterns (category 1) and networks with noticeable separate branching patterns (category 2). A distinct segmentation algorithm was designed for each category, involving common initial steps of contrast enhancement and median filtering to reduce noise and delineate CNV regions. Clinicians manually marked lesion locations for segmentation, and pixel-intensity-based measures, such as the BVD metric, quantified the proportion of blood vessels in CNV regions. The algorithm for category 2 patients demonstrated superior performance compared to the one for category 1.

Automated segmentation algorithms [6,10,34,35] offer notable advantages to clinicians, such as enabling rapid and accurate detection and quantification of CNV lesions in AMD patients. These approaches reduce clinicians’ efforts in interpreting complex OCTA images of retinal vascular layers. Despite their potential clinical benefits and innovative contributions, these algorithms have limitations, including the exclusion of some AMD patients from automated analysis in earlier studies [10,34]. In certain cases, patients were grouped based on CNV lesion topology or texture characteristics, leading to the development of distinct algorithms [35]. This grouping often stemmed from challenges such as graders’ inability to identify CNV regions or CNV lesions being insufficiently perceptible or fully contained within OCTA images [10,34,35]. Accurate detection of CNV areas in OCTA images remains essential for precise quantification over regions of interest.

Moreover, many automated segmentation methods [6,10,34,35] rely on expert clinicians to manually adjust the depth levels of OCTA imaging to capture optimal details of CNV lesion regions. While this adjustment can enhance image quality, it introduces potential bias, limiting the utility of the OCTA technique in providing automated segmentation of retinal vascular layers. These limitations hinder the clinical applicability and accuracy of most existing approaches for analysing AMD patients [6,10,34,35]. Consequently, the benefits of automated segmentation techniques and the broader adoption of OCTA imaging remain constrained by these challenges.

Image or texture classification tasks differ from image segmentation, as they aim to assign an entire image or texture region to a predefined category based on training samples. While several studies [6,10,34,35] focus on automating the segmentation of OCTA image textures for AMD, automated classification of OCTA images with AMD is underexplored. This gap arises due to the novelty of the OCTA imaging technique, the scarcity of labelled OCTA datasets for AMD, and the difficulty in obtaining healthy control samples essential for classification tasks. Despite these challenges, notable advancements in automated OCTA image classification for AMD include works by Vaghefi et al. [36] and Wang et al. [37].

The study by Vaghefi et al. [36] explored the integration of various ocular vascular imaging modalities (OCTA, OCT, and colour fundus photography (CFP)) to enhance dry AMD detection accuracy compared to single-modality analysis. CFP, unlike techniques such as FA and IGA, does not require a contrast agent, using white light to capture full-colour images of the retina [38]. The study involved 75 participants divided into three groups: young healthy (YH), old healthy (OH), and dry AMD patients. Each participant underwent multiple imaging techniques, including CFP, OCT, and OCTA, ensuring comprehensive data collection. The study used raw image data without pre-processing, with individual retinal and choroidal layers from OCTA identified and extracted automatically.

Vaghefi et al. [36] employed deep learning to develop and evaluate Convolutional Neural Networks (CNNs)-based image classification models using single, dual, and multimodal data combinations. Three designs were tested: single-modality CNNs trained separately on CFP, OCT, and OCTA; dual-modality CNNs combining OCT + OCTA and OCT + CFP; a multimodality CNN combining OCT + OCTA + CFP. These models classified participants into YH, OH, or dry AMD groups. Results showed that single-modality CNNs using CFP and OCTA data were most effective for detecting dry AMD, while the OCT-based CNN was better at identifying ageing groups (YH and OH). Diagnostic accuracy improved with multimodal data, with the multimodality CNN achieving near-perfect accuracy (99%).

The evaluation confirmed that combining imaging modalities significantly enhances diagnostic performance for dry AMD and ageing detection. Single-modality OCTA-based CNNs achieved 91% accuracy, dual-modality models (OCT + OCTA) reached 96%, and the multimodality CNN (OCT + OCTA + CFP) yielded 99% accuracy. Table 1 summarises the performance improvements across CNN designs, highlighting the added diagnostic value of integrating multimodal data. This underscores the potential of leveraging diverse imaging techniques for advancing retinal image analysis and understanding.

The study by Wang et al. [37] introduces a novel automated algorithm for identifying and segmenting CNV lesions in OCTA images, specifically addressing late-stage AMD (wet AMD). The algorithm integrates classification and segmentation tasks using two CNN models, which complement each other to classify OCTA images based on the presence of CNV lesions and segment these areas if present. The process begins by classifying OCTA images as CNV-positive or CNV-free, followed by segmenting CNV areas in the identified cases. The algorithm leverages diverse and information-rich OCTA datasets, including images from various retinal layers and volumetric data, ensuring robust training. Pre-processing steps, such as depth-level adjustments, layer subtraction, and manual annotation of CNV lesions by clinicians, were employed to construct accurate ground truth data.

The datasets comprised 1676 OCTA images from 607 eyes, including 117 CNV cases and 490 non-CNV controls. These datasets underwent rigorous pre-processing to ensure accurate segmentation and classification. In training, diverse representations of OCTA images were used, while testing relied on a single OCTA image of the outer retinal layer per eye, chosen for its clarity in visualising CNV lesions. The algorithm was evaluated with distinct datasets to avoid overlap between training and testing, ensuring unbiased results. The testing set included 50 CNV and 60 non-CNV eyes, reflecting the outer retinal layer’s prominence in detecting CNV lesions. The training set had 764 images from CNV eyes and 802 from non-CNV eyes, showcasing a broad representation of conditions.

Evaluation of the algorithm demonstrated exceptional performance. The classification achieved a sensitivity of 100% and specificity of 95%, with an AUC measure of 99%, indicating near-perfect diagnostic accuracy. The segmentation tasks were equally successful, producing precise blood vessel masks corresponding to CNV lesions. The robust performance underscores the algorithm’s potential for advancing OCTA image analysis, with its dual focus on classification and segmentation paving the way for improved diagnostic workflows in clinical settings.

From an image segmentation perspective, the automated algorithm by Wang et al. [37] demonstrated promising results in detecting CNV lesions in OCTA images, achieving a mean intersection over union (IOU) value of 0.88±0.10. This measure reflects the overlap accuracy between manually and automatically segmented regions, with values over 0.50 indicating reliable performance. In comparison, Liu et al. [10] achieved a mean IOU of 0.61±0.23 on the same dataset. These findings underscore the capability of automated segmentation schemes to facilitate precise lesion identification. Moreover, automated diagnostic algorithms, such as those proposed by Wang et al. [37] and Vaghefi et al. [36], offer significant clinical advantages by reducing clinician workload and minimising human errors, while maximising the potential of imaging modalities like OCTA in understanding conditions such as AMD.

Vaghefi et al. [36] highlighted the sensitivity of OCT, OCTA, and CFP imaging techniques to various ocular conditions, revealing nuanced insights like the higher sensitivity of OCT to ageing and CFP/OCTA to vascular pathologies such as AMD. Similarly, Wang et al. [37] demonstrated that automating CNV lesion segmentation improved patient care by streamlining tasks and identifying potential biomarkers for disease progression. However, both algorithms face challenges, including dependency on small, labelled datasets, which limits their generalisability. Rigorous evaluation using large datasets or cross-validation strategies is crucial for enhancing their robustness but is hindered by the computational demands and limited availability of labelled OCTA images due to the technique’s recent introduction.

The reliance on deep learning-based algorithms [36,37] introduces additional complexities, such as overfitting or underfitting due to inadequate datasets, and resource-intensive requirements like GPU hardware. While these challenges can be mitigated by combining imaging data from various modalities, as demonstrated by Vaghefi et al. [36], or creating binary classifications, as in Wang et al.’s [37] work, such approaches are not without limitations. For instance, diverse and messy data can obscure the identification of robust, representative features, and the pre-processing steps required, such as manually adjusting imaging depth and segmenting regions, introduce subjectivity and potential biases, complicating the results.

Despite the limitations, OCTA remains a valuable imaging modality, albeit with restricted availability of datasets for ocular conditions like AMD [36,37]. Current studies, including Wang et al. [37] and Vaghefi et al. [36], serve as proofs of concept, demonstrating the potential of automated algorithms to address clinical challenges. However, these algorithms require further validation with larger datasets to ensure reliability. The ideal solution under current constraints would involve developing algorithms that maintain high diagnostic accuracy with limited labelled data, enabling the effective utilisation of OCTA for AMD diagnosis and monitoring.

## 2. Materials and Methods

In this section, we will present the dataset used in this study and outline the proposed methodologies for AMD detection in OCTA images.

### 2.1. Image Data Description

The work presented in this paper makes use of OCTA image datasets that are captured from a 3 mm × 3 mm field of view that is centred around the fovea region. The dimensions of the OCTA images are 320×320 pixels. The study also utilises the automatically projected and segmented OCTA image datasets that show the typical ocular vascular texture appearance at four distinct retinal and choroidal depth level slabs or layers. The depth levels of OCTA images that show the various retinal and choroidal layers are unaltered and were extracted directly by the default-defined settings of ocular tissue depth levels measurements and segmentations of the OCTA imaging techniques used. This is to avoid the additional complexity of manual manipulations of adjusting and segmenting the OCTA images. The OCTA image datasets used in this research were provided by Manchester Royal Eye Hospital and Moorfields Eye Hospital.

**Figure 2:**
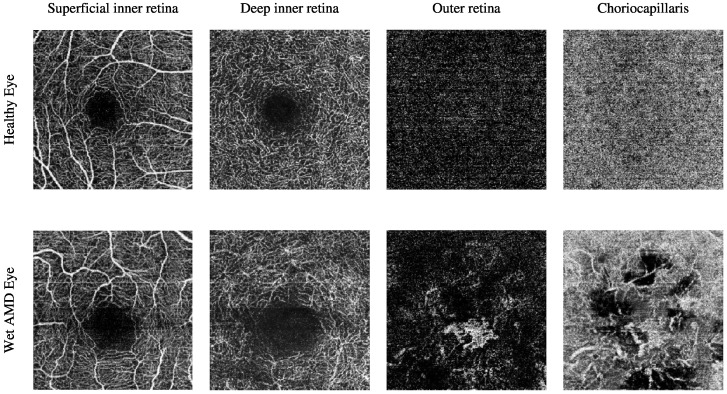
Visualising the various retinal vascular layers of two randomly selected eyes with different ocular conditions, specifically healthy and wet AMD eyes from the Manchester Royal Eye Hospital OCTA image dataset.

The Manchester Royal Eye Hospital OCTA image dataset comprises various subjects with healthy and late-stage AMD, i.e., wet AMD, eyes. The number of healthy eyes involved in this study is 33, while the number of wet AMD eyes is 23. Table 2 provides a detailed description of the number of OCTA images. Figure 2 demonstrates the textural appearance of the individual retinal vascular layers, namely the superficial inner retina, the deep inner retina, the outer retina and the choriocapillaris layers of randomly selected healthy and wet AMD eyes. The imaging platform used was Topcon Triton.

**Figure 3:**
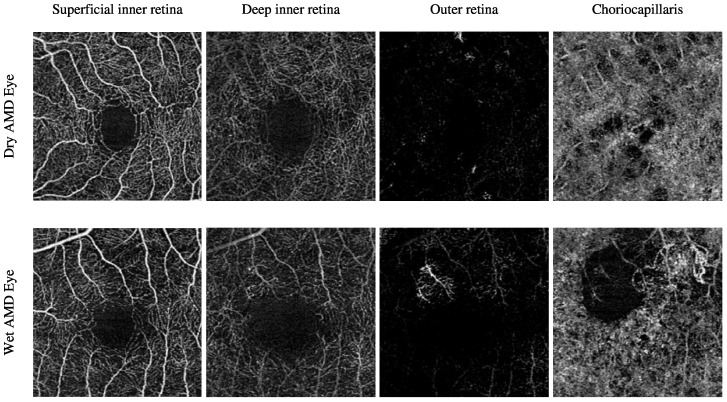
Demonstrating the various retinal vascular layers of two randomly selected eyes with different ocular conditions, specifically dry AMD and wet AMD eyes, from the Moorfields Eye Hospital OCTA image dataset.

The Moorfields Eye Hospital OCTA image dataset includes numerous subjects with different types of AMD, i.e., dry AMD and wet AMD, in their eyes. Other subjects with secondary CNV that share similar abnormal vascular characteristics of wet AMD are also included. They are denoted as secondary CNV because the causes of CNV regions are due to ophthalmic vascular pathologies other than AMD. The number of dry AMD, wet AMD, and secondary CNV eyes involved in this study is 79, 166, and 25, respectively. Table 3 provides a comprehensive description of the number of OCTA images. Figure 3 demonstrates the textural appearance of the individual retinal vascular layers, namely the superficial inner retina, the deep inner retina, the outer retina, and the choriocapillaris layers of randomly selected dry AMD and wet AMD eyes. The imaging platform used was ZEISS AngioPlex.

### 2.2. Description of Algorithms

To enable accurate and automated detection of AMD from OCTA images, two classification algorithms were developed. The first algorithm relies on whole local texture features, while the second utilises reduced local texture features transformed via the PCA technique.

#### 2.2.1. Classification Algorithm Based on Whole Local Texture Features

**Figure 4:**
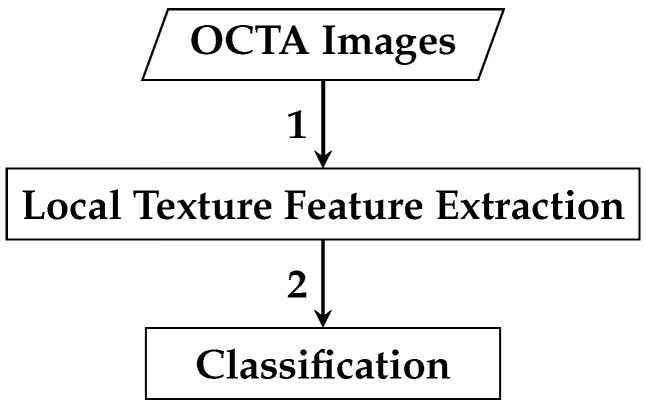
Outline of the automated OCTA image texture analysis algorithm that is proposed for AMD detection by the means of image classification. The analysis procedure begins with taking the OCTA images as input where the local texture features extraction step takes place first to quantify the textural appearance of OCTA images. Following this step, the classification is then performed over the extracted local texture features that quantify the various OCTA images to classify them based on the ocular conditions they represent e.g., dry AMD, wet AMD, and healthy.

The framework of the first automated classification algorithm proposed for AMD detection based on whole local texture features involves two main steps. The first step is the local texture feature extraction, while the second step is the classification. Figure 4 demonstrates a brief overview of the analysis pipeline that is followed by the automated OCTA image classification algorithm proposed.

##### Local Texture Feature Extraction

The illumination and rotation-invariant uniform $LBP^{2riu}$ texture descriptor proposed by Ojala et al. [27] is a promising choice for texture analysis in OCTA images. Its invariance to illumination and rotation changes allows it to generate discriminative features, making it effective for datasets of varying sizes. These attributes render it suitable for capturing texture features associated with AMD in OCTA images. Studies such as Liu et al. [39] demonstrate $LBP^{2riu}$’s robustness under challenging conditions, including illumination and rotation variations in classification and segmentation tasks [40]. Given that OCTA imaging produces greyscale images highlighting blood vessels and flows, $LBP^{2riu}$ is advantageous for quantifying monochrome textures [41]. Its low computational complexity and minimal parameter requirements further enhance its suitability for OCTA texture analysis.

To validate $LBP^{2riu}$, two additional descriptors, the generic local binary pattern (LBP) by Ojala et al. [27,42,43] and the binary robust independent elementary features (BRIEF) by Calonder et al. [44,45], are tested. Both share advantages with $LBP^{2riu}$, including low computational complexity, minimal parameter tuning, and resilience to uneven illumination, with BRIEF also robust to minor rotations [44,45]. Their effectiveness in medical image texture analysis is evidenced in various studies [38,46,47,48,49,50,51,52,53,54]. Evaluating these descriptors alongside $LBP^{2riu}$ confirms its suitability and addresses the limited prior research on automated AMD detection in OCTA images [36,37].

The generic LBP texture descriptor, including variants such as $LBP^{2riu}$, relies on fine-tuning two key parameters, *p* and *r*, where p>1 and r>0. The parameter *p* determines the number of sampling pixels in an evenly spaced circularly symmetric neighbourhood centred on a single central pixel, while *r* defines the radius, i.e., the distance from the central pixel to the *p* sampling pixels. The values of these *p* sampling pixels, along with the central pixel, are used by $LBP_{p,r}$ and $LBP_{p,r}^{2riu}$ descriptors to characterise local texture features in OCTA images.

The OCTA imaging technique generates two-dimensional greyscale images of ocular vascular layers, where *p* sampling pixels and the central pixel represent grey levels *g*. For a two-dimensional greyscale OCTA image (x,y), the grey levels of *p* sampling pixels are denoted as $g_{n}$ at $\left(x_{n},y_{n}\right)$, with n=(0,1,2,…,p−1), and the central pixel’s grey level as $g_{c}$ at $\left(x_{c},y_{c}\right)$. When the central pixel $g_{c}$ at $\left(x_{c},y_{c}\right)$ is (0,0), the coordinates of *p* sampling pixels $g_{n}$ at $\left(x_{n},y_{n}\right)$ are provided by (−rsin(2πn/p),rcos(2πn/p)). If any *p* sampling pixel $g_{n}$ does not align with a pixel in the OCTA image, its location is estimated via bilinear interpolation.

Estimating the values and coordinates of all *p* sampling pixels can be done in any ordering direction, e.g., clockwise or anticlockwise, starting from any location in the circularly symmetric neighbourhood of *p* sampling pixels. For instance, the starting sampling pixel $g_{0}$ can be the one to the right or left of the central pixel $g_{c}$. However, the ordering direction and the starting pixel $g_{0}$ must remain consistent. In this study, $g_{0}$ is always assigned to the pixel to the right of $g_{c}$, with the ordering direction being clockwise for all regions or pixels in the OCTA image and any subsequent OCTA images measured.

Once the values and coordinates of all *p* sampling pixels $g_{n}$ at $\left(x_{n},y_{n}\right)$ are estimated, the $LBP_{p,r}$ and $LBP_{p,r}^{2riu}$ descriptors typically characterise OCTA image textures by considering only the signs of differences between the grey level of the central pixel $g_{c}$ and the neighbourhood of *p* sampling pixels $g_{n}$ rather than their exact grey levels, such that $s\left(g_{0}-g_{c}\right),s\left(g_{1}-g_{c}\right),\ldots ,s\left(gp-1-g_{c}\right)$. These signs are then passed through a thresholding operation s(x), i.e., a binary test, as defined by Function (1).

(1) $$\begin{array}{c} s\left(x\right)=\left\{\begin{array}{cc} 1 & \;\mathrm{if}\;x\geq 0\\ 0 & \;\mathrm{Otherwise} \end{array}\right. \end{array}.$$

The thresholding operation s(x) in Function (1) constructs different *p*-bit binary numbers or binary strings, i.e., various local binary patterns. However, the individual binary numbers, i.e., 0 and 1, in the local binary patterns of different regions visited, i.e., central pixels, are typically weighted by powers of two, i.e., $2^{n}$, and summed to convert the binary strings into decimal representations. These decimal representations are then used to label the individual regions, i.e., central pixels, being visited and measured. The mathematical expression of the generic $LBP_{p,r}$ texture descriptor for characterising the textural appearance around any random region, i.e., an arbitrary central pixel at $\left(x_{c},y_{c}\right)$ in the two-dimensional OCTA images, can thus be formally defined by Equation (2).

(2) $$\begin{array}{c} LBP_{p,r}\left(x_{c},y_{c}\right)=\sum_{n=0}^{n=p-1}s\left(g_{n}-g_{c}\right)\;2^{n}. \end{array}$$

In practice, the generic $LBP_{p,r}$ texture descriptor in Equation (2) generates $2^{p}$ possible $LBP_{p,r}$ patterns, i.e., $2^{p}$ distinct decimal values. These patterns capture specific spatial structures of local texture features, e.g., corners and edges, in image textures. In this study, the $LBP_{p,r}$ descriptor is applied densely to every pixel of OCTA images, generating various $LBP_{p,r}$ pattern combinations. The local texture features measured by the $LBP_{p,r}$ descriptor are summarised into a single histogram per OCTA image, with approximately $2^{p}$ bins corresponding to all potential $LBP_{p,r}$ patterns, as defined in Equation (3).

(3) $$LBP_{H}=\left\{h_{0},h_{1},h_{2},\ldots ,h_{2^{p}-1}\right\},$$

where $LBP_{H}$ represents the histogram, $h_{i}$ denotes the frequency of the *i*-th bin, and $2^{p}$ is the total number of bins corresponding to the distinct decimal values generated by the generic $LBP_{p,r}$ texture descriptor. Each bin represents a unique $LBP_{p,r}$ pattern.

**Figure 5:**
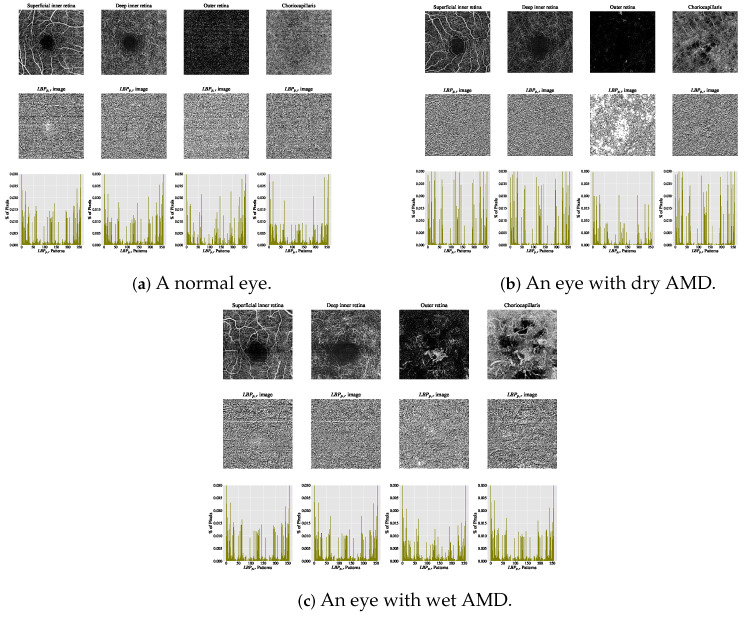
Visualising various OCTA images with their corresponding $LBP_{p,r}$ images and histograms. (**a**) Demonstrates a normal eye. (**b**) Shows an eye with dry AMD. (**c**) Displays an eye with wet AMD.

The estimated histograms of individual OCTA images construct the feature vectors used for automated OCTA image texture analysis via image classification. Figure 5 presents examples of OCTA images from four ocular vascular layers, their corresponding encoded texture structures ($LBP_{p,r}$ images), and feature vectors (histograms) for three eye conditions: healthy, dry AMD, and wet AMD. In each figure, the texture of each OCTA image is measured with r=1 and p=8 to illustrate how the $LBP_{p,r}$ descriptor quantifies the texture of each condition in the OCTA images.

Although the generic $LBP_{p,r}$ texture descriptor is invariant to illumination changes, it is highly sensitive to orientation variations in image textures. This occurs because changes in image orientation alter the values and coordinates of all *p* sampling pixels $g_{n}$ at $\left(x_{n},y_{n}\right)$ along the perimeter of the circularly symmetric neighbourhood. Since the starting sampling pixel, $g_{0}$, is always assigned to the pixel immediately to the right of the central pixel $g_{c}$, rotating an $LBP_{p,r}$ pattern results in a different decimal value. However, this does not affect $LBP_{p,r}$ patterns composed entirely of 0s or 1s, as these remain unchanged regardless of the texture image’s rotation.

The $LBP_{p,r}^{2riu}$ texture descriptor mitigates sensitivity to rotation changes and enhances tolerance to illumination variations in texture images by combining the rotation invariant $LBP_{p,r}^{ri}$ and the uniform $LBP_{p,r}^{2u}$ versions of $LBP_{p,r}$. As implied by its name, $LBP_{p,r}^{ri}$ is highly robust to image rotation, achieved by mapping (circularly rotating) each $LBP_{p,r}$ pattern to its minimum representation. The $LBP_{p,r}^{ri}$ descriptor mathematically estimates rotation invariant local texture features around any arbitrary central pixel $\left(x_{c},y_{c}\right)$ in different OCTA images using the following Equation (4).

(4) $$\begin{array}{c} LBP_{p,r}^{ri}\left(x_{c},y_{c}\right)=min\left\{ROR\left(LBP_{p,r}\left(x_{c},y_{c}\right),n\right)|n=\left(0,1,2,3,\ldots ,p-1\right)\right\}. \end{array}$$

The $ROR(LBP_{p,r}\left(x_{c},y_{c}\right),n)$ function in Equation (4) performs a circular bit-wise right shift, or right rotation, on the *p*-bit binary numbers or $LBP_{p,r}$ patterns by *n* steps. This operation rotates the neighbourhood of *p* sampling pixels clockwise so that the least significant bit, starting from $g_{0}$, is 1. For instance, $LBP_{p,r}$ patterns with p=8 bits, such as 00000010, 00000100, and 00001000, are all mapped to the minimum $LBP_{p,r}$ pattern, 00000001, by 1, 2, and 3 steps, respectively.

The uniform $LBP_{p,r}^{2u}$ texture descriptor offers advantages such as statistical robustness and the stability of derived uniform local texture features, i.e., the uniform $LBP_{p,r}^{2u}$ patterns. These patterns are shown to be less affected by and more resistant to image noise, as evidenced by numerous studies [27,41,55]. The descriptor calculates the number of bitwise transitions from 1 to 0 or vice versa in the $LBP_{p,r}$ patterns. The uniformity measure of the $LBP_{p,r}^{2u}$ texture descriptor is formally defined by Equation (5).

(5) $$\begin{array}{c} u\left(LBP_{p,r}\right)=\left|s\left(g_{p-1}-g_{c}\right)-s\left(g_{0}-g_{c}\right)\right|\\ +\sum_{n=1}^{n=p-1}\left|s\left(g_{n}-g_{c}\right)-s\left(g_{n-1}-g_{c}\right)\right|. \end{array}$$

Based on the uniformity pattern measure $u(LBP_{p,r})$ defined in Equation (5), an $LBP_{p,r}$ pattern is classified as a uniform $LBP_{p,r}^{2u}$ pattern if the uniformity measure, i.e., the number of bitwise transitions, is at most two. For instance, patterns such as 00000000 (zero transitions), 00000001 (one transition), and 00111000 (two transitions) are uniform $LBP_{p,r}^{2u}$ patterns as they involve no more than two 1/0 or 0/1 changes. Conversely, patterns like 11000101 (four transitions), 01010011 (five transitions), and 01101010 (six transitions) are not uniform $LBP_{p,r}^{2u}$ patterns as they exceed the two-transition limit.

Given the notable advantages, such as statistical stability and robustness to image noise, provided by the previously mentioned texture descriptors, i.e., $LBP_{p,r}^{ri}$ and $LBP_{p,r}^{2u}$, the $LBP_{p,r}^{2riu}$ is a highly effective texture descriptor. The mathematical expression of the $LBP_{p,r}^{2riu}$, used to estimate rotation-invariant uniform local texture features around any arbitrary central pixel at $\left(x_{c},y_{c}\right)$ in two-dimensional OCTA images, is formally presented in Equation (6).

(6) $$\begin{array}{c} LBP_{p,r}^{2riu}\left(x_{c},y_{c}\right)=\left\{\begin{array}{cc} \sum_{n=0}^{n=p-1}s\left(g_{n}-g_{c}\right) & \;\mathrm{if}\;u(LBP_{p,r})\leq 2\\ p+1 & \;\mathrm{Otherwise} \end{array}\right. \end{array}.$$

By definition, only p+1 rotation invariant uniform local binary patterns, i.e., rotation invariant uniform $LBP_{p,r}^{2riu}$ patterns, can exist in a circularly symmetric neighbourhood of *p* sampling pixels. The $LBP_{p,r}^{2riu}$ descriptor in Equation (6) assigns a unique integer label from 0 to *p* for each rotation invariant uniform $LBP_{p,r}^{2riu}$ pattern based on the number of ones (“1 s” bits) in the pattern, while non-uniform $LBP_{p,r}^{2riu}$ patterns are collectively assigned a single label, p+1.

The $LBP_{p,r}^{2riu}$ texture descriptor generates p+2 distinctive output labels, quantifying local texture structures like corners, edges, and patterns in OCTA images. In this study, it is densely applied to every pixel of the OCTA images, constructing various rotation-invariant uniform $LBP_{p,r}^{2riu}$ patterns.

The local texture features measured by the $LBP_{p,r}^{2riu}$ descriptor are summarised using a single histogram per OCTA image. Each histogram, containing approximately p+2 bins, represents the discrete distribution of various labels or values. These labels correspond to all possible rotation-invariant uniform and non-uniform $LBP_{p,r}^{2riu}$ patterns, as defined in Equation (7).

(7) $$\begin{array}{cc} LBP_{H}^{2riu} & =\left\{h_{0},h_{1},h_{2},\ldots ,h_{p+1}\right\}, \end{array}$$

where $LBP_{H}^{2riu}$ represents the histogram, $h_{i}$ denotes the frequency of the *i*-th bin, and p+2 is the total number of bins corresponding to the distinct labels or values generated by the $LBP_{p,r}^{2riu}$ texture descriptor. Each bin represents a unique rotation-invariant uniform or non-uniform pattern.

**Figure 6:**
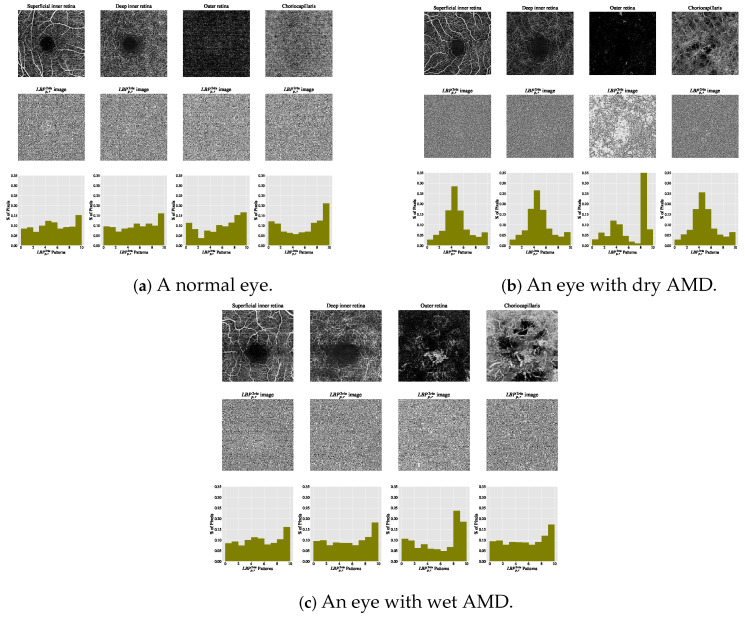
Visualising various OCTA images with their corresponding $LBP_{p,r}^{2riu}$ images and histograms. (**a**) A normal eye. (**b**) An eye with dry AMD. (**c**) An eye with wet AMD.

The estimated histograms of individual OCTA images construct feature vectors forming the basis for automated OCTA image texture analysis via image classification. Figure 6 shows examples of OCTA images from four ocular vascular layers with their encoded texture structures, i.e., the $LBP_{p,r}^{2riu}$ images, and corresponding histograms for three conditions: healthy, dry AMD, and wet AMD. In each figure, the texture of each OCTA image was measured with r=1 and p=8, highlighting how the $LBP_{p,r}^{2riu}$ descriptor quantifies different eye conditions in OCTA images.

The $BRIEF_{S,n}$ texture descriptor relies on two key parameters: *S* and *n*. The value of *S* determines the size of image patches *P*, i.e., S×S pixels, while *n* specifies the number of sampling pixels, i.e., *n* sampling pixel pairs, within each patch *P*. Once these values, *S* and *n*, are defined, the $BRIEF_{S,n}$ descriptor quantifies OCTA image textures by evaluating the signs of differences between grey-level values of the *n* sampling pixel pairs rather than their exact grey levels. These signs of differences within each patch *P* of size S×S pixels are then passed through a thresholding operation τ(P:X,Y), a binary test step, as defined by Function (8).

(8) $$\begin{array}{c} \tau \left(P:X,Y\right)=\left\{\begin{array}{cc} 1 & \;\mathrm{if}\;(P(X)-P(Y))\;>\;A\;threshold\;value\\ 0 & \;\mathrm{Otherwise} \end{array}\right. \end{array},$$

where P(X) and P(Y) represent the grey-level values of *n* pixel pairs in image patch *P*, sampled at random locations *X* and *Y*. As demonstrated in prior studies [38,46,47], the random sampling strategy ensures an equal distribution of *X* and *Y* across the image patch *P*, allowing binary tests to extend near the patch border. Consequently, important texture regions, such as CNV areas, can be effectively visited and measured regardless of their location in different OCTA images.

The threshold value is estimated based on the image noise level, i.e., the noise standard deviation (σ). As in previous studies [38,46,47], σ is calculated by blurring an OCTA image with a Gaussian filter and subtracting the filtered image from the original, yielding a resultant image containing only the noise signal. σ is then estimated from this image. In this study, the threshold is set to three times σ (3σ), which achieves good classification performance. Higher values, e.g., 4σ, showed a slight drop in accuracy [38]. As the OCTA data are in greyscale, 3σ roughly corresponds to a grey level between four and six.

The thresholding test operation τ(P:X,Y) in Function (8) generates *n*-bit binary numbers or strings, representing local binary patterns. The binary digits (0 and 1) in these patterns, derived from image patches *P*, are weighted by powers of two, $2^{i-1}$ where (1≤i≤n), and summed to convert the strings into decimal values. These decimal values label the respective regions (image patches *P*) being analysed. The $BRIEF_{S,n}$ texture descriptor is formally defined in Equation (9).

(9) $$\begin{array}{c} BRIEF_{S,n}P=\sum_{1\leq i\leq n}\tau \left(P:X_{i},Y_{i}\right)\;2^{i-1}. \end{array}$$

In practice, the $BRIEF_{S,n}$ texture descriptor in Equation (9) can generate $2^{n}$ potential $BRIEF_{S,n}$ pattern combinations, i.e., $2^{n}$ distinct decimal values. These patterns measure the spatial structure of local texture features in various images. In this study, the $BRIEF_{S,n}$ descriptor is densely applied to every region, extracting a patch *P* from each pixel of the OCTA images to construct $BRIEF_{S,n}$ combinations. The measured texture features are summarised by a single histogram for each OCTA image, comprising approximately $2^{n}$ bins to accumulate decimal values of all potential $BRIEF_{S,n}$ patterns, as defined in Equation (10).

(10) $$BRIEF_{H}=\left\{h_{0},h_{1},h_{2},\ldots ,h_{2^{n}-1}\right\},$$

where $BRIEF_{H}$ represents the histogram, $h_{i}$ denotes the frequency of the *i*-th bin, and $2^{n}$ is the total number of bins corresponding to the distinct decimal values generated by the $BRIEF_{S,n}$ texture descriptor. Each bin represents a unique $BRIEF_{S,n}$ pattern.

**Figure 7:**
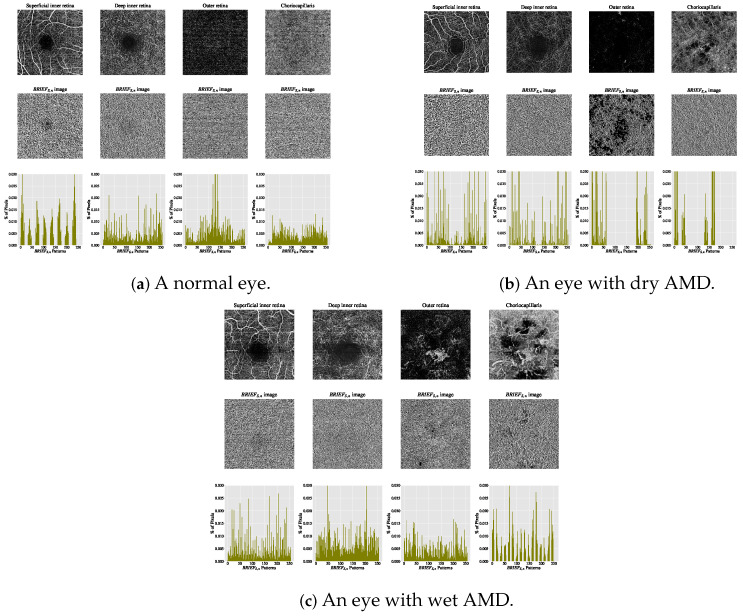
Visualising various OCTA images with their corresponding $BRIEF_{S,n}$ images and histograms. (**a**) A normal eye. (**b**) An eye with dry AMD. (**c**) An eye with wet AMD.

The estimated histograms of individual OCTA images form the feature vectors underpinning automated OCTA texture analysis through image classification. Figure 7 illustrates examples of OCTA images across four ocular vascular layers, their encoded texture structures ($BRIEF_{S,n}$ images), and feature vectors (histograms) for three eye conditions: healthy, dry AMD, and wet AMD. In each figure, the texture of each OCTA image was measured empirically with S=5 and n=8, showcasing how the $BRIEF_{S,n}$ texture descriptor quantifies these conditions.

The rationale for employing texture descriptors such as $LBP_{p,r}^{2riu}$, $LBP_{p,r}$, and $BRIEF_{S,n}$ lies in their capacity to capture microstructural variations in OCTA images that are closely linked to pathological changes in AMD. In both dry and wet AMD, OCTA imaging reveals hallmark features such as choriocapillaris rarefaction, geographic atrophy, and choroidal neovascularisation (CNV), which manifest as localised disruptions or irregularities in vascular texture [6,37]. These changes alter the local intensity patterns and introduce distinct structural signatures, such as edges, discontinuities, and uniform or non-uniform patch distributions, that are effectively captured by $LBP_{p,r}^{2riu}$, $LBP_{p,r}$, and $BRIEF_{S,n}$ patterns.

Specifically, $LBP_{p,r}^{2riu}$ descriptor captures repetitive and orientation-invariant structures such as vessel loops or homogeneous regions of dropout, which are common in areas of geographic atrophy. Non-uniform patterns, by contrast, are more likely to occur in regions with active CNV, where the chaotic vasculature introduces irregular texture patterns. The $BRIEF_{S,n}$ descriptor, which operates on patch-wise intensity comparisons, is sensitive to subtle changes in microvascular density and texture randomness, thereby capturing features related to early capillary dropout or neovascular changes. By summarising these local patterns into histograms, the resulting feature vectors provide a discriminative representation of AMD-induced morphological disruptions in the vasculature.

From a feature engineering perspective, the local texture feature extraction step is highly effective as it applies two distinct approaches to OCTA image texture patterns: structural and statistical. The structural approach characterises micro-structures in the texture, e.g., edges, corners, and lines, using $LBP_{p,r}^{2riu}$, $LBP_{p,r}$, and $BRIEF_{S,n}$ descriptors. The statistical approach estimates the distribution of these micro-structures in histograms, constructing feature vectors essential for automated OCTA image texture analysis via image classification.

##### Classification

Once feature vectors for individual OCTA images are constructed, the classification step aims to categorise eyes into predefined classes of eye conditions, such as healthy, wet AMD (CNV), or dry AMD (non-CNV). To achieve this, two machine learning algorithms (support vector machine (SVM) and K-nearest neighbour (KNN)) are employed and evaluated. These classifiers were selected due to their widespread use in texture and medical image analysis, where they have consistently demonstrated effective performance with representative texture features [39].

The choice of SVM and KNN is further supported by their strong theoretical foundations and established effectiveness in texture-based and medical imaging tasks [39,56,57]. SVM is particularly well-suited to high-dimensional feature spaces and performs effectively when class boundaries are complex or non-linear. Additionally, SVM’s reliance on a small subset of training samples, i.e., support vectors, makes it inherently more robust to class imbalance, which is commonly observed in medical datasets.

KNN, by contrast, is a simple, non-parametric algorithm valued for its strong interpretability, an important trait in clinical settings. Its classification performance relies heavily on the quality of the input features, making it a useful benchmark for evaluating the discriminative power of the texture representations developed in this study.

#### 2.2.2. Classification Algorithm Based on Reduced Local Texture Features

**Figure 8:**
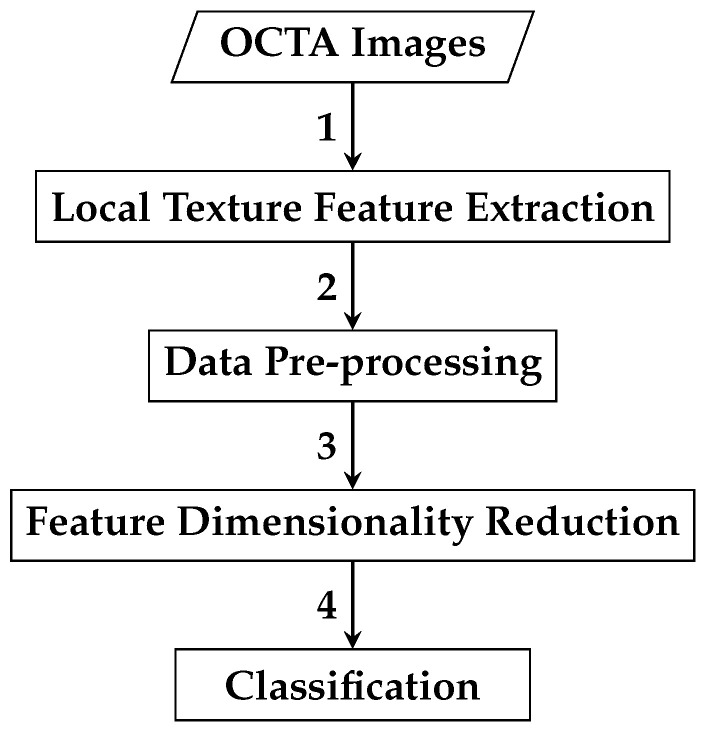
An outline of the automated OCTA image texture analysis algorithm that is proposed for AMD detection by the means of image classification based on reduced local texture features. The analysis procedure begins with taking the OCTA images as an input where the local texture feature extraction step takes place first to quantify the textural appearance of OCTA images. A data pre-processing step is then followed, which involves applying data transformation techniques, specifically data centring and scaling techniques to the local texture features extracted. The following step is the feature dimensionality reduction that applies the PCA technique to decorrelate the extracted local texture features generating a representative and reduced set of local texture features. Finally, the classification step is followed to perform classifications using the reduced local texture features which measure the various OCTA images to classify them based on the ocular conditions they represent, e.g., dry AMD, wet AMD, and healthy.

The second automated classification algorithm proposed follows a pipeline similar to that in Section 2.2.1, with two additional steps: data pre-processing and feature dimensionality reduction. Thus, the framework for AMD detection based on reduced local texture features comprises four steps: local texture feature extraction, data pre-processing, feature dimensionality reduction, and classification. Figure 8 demonstrates a brief overview of the analysis pipeline that is followed by the automated OCTA image classification algorithm proposed.

##### Local Texture Feature Extraction

The goal of this local texture feature extraction step is to analyse the textural appearance of OCTA images under different ocular conditions: dry AMD, wet AMD, and healthy. Three texture descriptors, $LBP_{p,r}^{2riu}$, $LBP_{p,r}$, and $BRIEF_{S,n}$, are evaluated individually, following the same approach as in local texture feature extraction in the earlier classification algorithm Section 2.2.1. This process generates histograms (feature vectors) summarising the distribution of local texture patterns in OCTA images.

##### Data Pre-Processing

Once the feature vectors are constructed in the previous step, local texture feature extraction, in Section 2.2.2, the data pre-processing step applies two transformation techniques, data centring and scaling, to the whole local texture features. This ensures that all local texture features contribute equally to estimating the reduced feature vectors in the subsequent dimensionality reduction step, feature dimensionality reduction, in Section 2.2.2.

The motivation for employing these data transformation techniques is the variability in textural appearance across diseased OCTA images. In some cases, vascular abnormalities dominate the diseased images, while in others, they resemble healthy OCTA images. Transforming the whole local texture features of original feature vectors to comparable scales reduces the bias of the PCA technique toward local texture features with high range values during the estimation of reduced feature vectors.

As two data transformation techniques, i.e., data centring and scaling, are applied to the original feature vectors, the complete original feature vectors comprising whole local texture features of individual OCTA images can be represented as an N×F data matrix *D* as follows:

$$D_{N\times F}=\left[\begin{array}{cccc} x_{\left(11\right)} & x_{\left(12\right)} & \ldots & x_{\left(1F\right)}\\ x_{\left(21\right)} & x_{\left(22\right)} & \ldots & x_{\left(2F\right)}\\ \vdots & \vdots & \vdots & \vdots \\ x_{\left(N1\right)} & x_{\left(N2\right)} & \ldots & x_{\left(NF\right)} \end{array}\right],$$

where *x* denotes the local texture features, the subscript *N* represents the total number of OCTA images, and *F* indicates the total number of local texture features. In the data matrix *D*, *N* rows correspond to different OCTA images, and *F* columns represent various local texture features. After applying data centring and scaling, the mean μ and standard deviation σ of each column, i.e., individual *F* local texture features, are computed from *D*. The mean μ of each *F* feature, i.e., each column in *D*, is calculated using the following Formula (11):

(11) $$\begin{array}{c} \mu_{F}=\frac{1}{N}\sum_{i=1}^{N}x_{\left(iF\right)} \end{array}.$$

Therefore, the standard deviation σ of each *F* local texture feature, i.e., every single column in the data matrix *D*, can be mathematically calculated by the following Formula (12):

(12) $$\begin{array}{c} \sigma_{F}=\sqrt{\frac{\sum_{i=1}^{N}{\left(x_{\left(iF\right)}-\mu_{F}\right)}^{2}}{N}} \end{array}.$$

Once the $\mu_{F}$ and $\sigma_{F}$ of all *F* local texture features (i.e., all columns in the data matrix *D*) are estimated, each $x_{\left(iF\right)}$ is replaced by $\frac{x_{\left(iF\right)}-\mu_{F}}{\sigma_{F}}$ in *D*. This process yields a standardised data matrix SD, expressed as follows:

$$SD_{N\times F}=\left[\begin{array}{cccc} \frac{x_{\left(11\right)}-\mu_{1}}{\sigma_{1}} & \frac{x_{\left(12\right)}-\mu_{2}}{\sigma_{2}} & \ldots & \frac{x_{\left(1F\right)}-\mu_{F}}{\sigma_{F}}\\ \frac{x_{\left(21\right)}-\mu_{1}}{\sigma_{1}} & \frac{x_{\left(22\right)}-\mu_{2}}{\sigma_{2}} & \ldots & \frac{x_{\left(2F\right)}-\mu_{F}}{\sigma_{F}}\\ \vdots & \vdots & \vdots & \vdots \\ \frac{x_{\left(N1\right)}-\mu_{1}}{\sigma_{1}} & \frac{x_{\left(N2\right)}-\mu_{2}}{\sigma_{2}} & \ldots & \frac{x_{\left(NF\right)}-\mu_{F}}{\sigma_{F}} \end{array}\right].$$

##### Feature Dimensionality Reduction

Once the standardised data matrix SD, comprising whole local texture features of individual OCTA images with various ocular vascular conditions related to AMD and healthy cases, is constructed during data pre-processing in Section 2.2.2, the feature dimensionality reduction task decorrelates the individual local texture features of the original vectors using the PCA technique. Applying PCA generates a transformed set of reduced local texture features, i.e., reduced feature vectors, that aim to retain the majority of the significant information from the original feature vectors.

As the standardised data matrix SD is constructed, the initial step in the PCA technique involves transposing SD (flipping it over its diagonal) to produce its transpose, denoted by $SD^{T}$. The transposed matrix $SD^{T}$ is expressed as follows:

$$SD_{F\times N}^{T}=\left[\begin{array}{cccc} x_{\left(11\right)} & x_{\left(12\right)} & \ldots & x_{\left(1N\right)}\\ x_{\left(21\right)} & x_{\left(22\right)} & \ldots & x_{\left(2N\right)}\\ \vdots & \vdots & \vdots & \vdots \\ x_{\left(F1\right)} & x_{\left(F2\right)} & \ldots & x_{\left(FN\right)} \end{array}\right].$$

Once the transposed data matrix $SD^{T}$ is constructed, the next step is to compute the covariance matrix Σ using the standardised data matrix SD and its transpose, $SD^{T}$. For standardised data (mean = zero, standard deviation = one), the covariance matrix Σ is estimated by Equation (13):

(13) $$\begin{array}{c} \Sigma =SD^{T}SD\;N^{-1}=\frac{1}{N}\sum_{i=1}^{N}{x_{\left(i\right)}}^{T}x_{\left(i\right)} \end{array}.$$

The covariance matrix Σ has dimensions F×F. Once Σ is constructed, the next step is to estimate the principal components (PCs) by computing the eigenvectors ϕ and corresponding eigenvalues λ of Σ. The total variance (TV) in the original data is the sum of all *F* eigenvalues λ of Σ, as given in Equation (14):

(14) $$\begin{array}{c} TV=\sum_{j=1}^{F}\lambda_{j} \end{array}.$$

Hence, the proportion of the variance ($PV_{i}$) that the $i^{th}$ eigenvector ϕ is accounted for can be estimated by the following Equation (15):

(15) $$\begin{array}{c} PV_{i}=\frac{\lambda_{i}}{\sum_{j=1}^{F}\lambda_{j}} \end{array}.$$

The *F* eigenvalues λ are sorted in descending order, i.e., $\left(\lambda_{j}\geq \lambda_{j+1}\right)$. A set Φ of *K* eigenvectors ϕ, where $\Phi =[\phi_{1},\phi_{2},\ldots ,\phi_{K}]$ and typically K<F, is selected from the covariance matrix Σ based on the *K* largest eigenvalues λ to reduce the data dimensions. The eigenvector $\phi_{1}$ with the greatest eigenvalue $\lambda_{1}$ represents the first PC, the direction of maximum variation, while $\phi_{2}$, corresponding to $\lambda_{2}$, represents the second PC, orthogonal to the first, and so on.

The *K* eigenvectors ϕ corresponding to the *K* largest eigenvalues λ are typically chosen to retain a predefined proportion of the total variance, e.g., 95 for 95%. Thus, the set Φ of *K* eigenvectors ϕ retaining 95% of the total variance can be formed by summing the proportion of variance ($PV_{i}$) explained by the *K* largest eigenvalues λ, as shown in Equation (16):

(16) $$\begin{array}{c} \sum_{i=1}^{K}PV_{i}\geq 95 \end{array}.$$

Once the *K* eigenvectors ϕ with the *K* largest eigenvalues λ are selected, explaining the chosen variance proportion (e.g., 95 for 95% of the original data variance), the selected set Φ of *K* eigenvectors ϕ forms the set of PCs used to generate new transformed features, replacing the original local texture features. The final step in PCA involves projecting the original data into a reduced feature subspace by multiplying it with the selected set Φ of *K* eigenvectors ϕ. This step constructs the reduced feature vectors representing the transformed set of local texture features for the individual OCTA images.

In this work, the new reduced feature vectors, constructed during the feature dimensionality reduction step, retained 95% of the variance in the original data, a commonly used threshold when applying the PCA technique. The local texture features generated by the three texture descriptors, $LBP_{p,r}^{2riu}$, $LBP_{p,r}$, and $BRIEF_{S,n}$, are transformed into reduced features and denoted as $LBP_{p,r}^{2riuPCA}$, $LBP_{p,r}^{PCA}$, and $BRIEF_{S,n}^{PCA}$, respectively, with the superscript ^*PCA*^ indicating the application of PCA.

##### Classification

Once the reduced feature vectors of individual OCTA images are constructed, two machine learning classifiers, SVM and KNN, are tested for image classification, similar to the classification step described in the Classification section in Section 2.2.1.

## 3. Results

The evaluation of the algorithms in Section 2.2.1 and Section 2.2.2 was conducted on the diverse OCTA image datasets outlined in Section 2.1. This evaluation framed the problem as binary image classification on OCTA data from Manchester Royal Eye Hospital and Moorfields Eye Hospital. For Manchester, classification distinguished healthy from wet AMD. For Moorfields, it differentiated dry AMD from wet AMD. Additionally, a further binary classification on Moorfields data distinguished CNV (wet AMD plus secondary CNV) from non-CNV (dry AMD), as secondary CNV images share vascular abnormalities with wet AMD.

Let $LBP_{H}^{2riu}$, $LBP_{H}$, and $BRIEF_{H}$ represent the individual feature vectors extracted from OCTA images for each distinct ocular vascular layer, using the texture descriptors $LBP_{p,r}^{2riu}$, $LBP_{p,r}$, and $BRIEF_{S,n}$, respectively, as defined earlier. Consequently, for all conducted experiments, binary image classifications were performed as follows:

1. Based on the individual layer feature vector, for a single ocular vascular layer $V_{i}$, the feature vector $F_{V_{i}}$ is directly represented by the following histogram:
  $$F_{V_{i}}=\left\{\begin{array}{cc} LBP_{H}^{2riu} & \mathrm{if}\;LBP_{p,r}^{2riu}\;\mathrm{is}\;\mathrm{used}\\ LBP_{H} & \mathrm{if}\;LBP_{p,r}\;\mathrm{is}\;\mathrm{used}\\ BRIEF_{H} & \mathrm{if}\;BRIEF_{S,n}\;\mathrm{is}\;\mathrm{used} \end{array}\right..$$
2. Based on concatenating two layers, for two ocular vascular layers $V_{i}$ and $V_{j}$, the concatenated feature vector $F_{V_{i},V_{j}}$ is provided by the following:
  $$F_{V_{i},V_{j}}=F_{V_{i}}⊕F_{V_{j}},$$
  where ⊕ denotes the concatenation operation.
3. Based on concatenating three layers, for three ocular vascular layers $V_{i}$, $V_{j}$, and $V_{k}$, the concatenated feature vector $F_{V_{i},V_{j},V_{k}}$ is as follows:
  $$F_{V_{i},V_{j},V_{k}}=F_{V_{i}}⊕F_{V_{j}}⊕F_{V_{k}}.$$
4. Based on concatenating all layers, for all ocular vascular layers $V_{1},V_{2},V_{3},V_{4}$ (namely, the superficial inner retina, the deep inner retina, the outer retina, and the choriocapillaris), the global feature vector $F_{global}$ is as follows:
  $$F_{global}=F_{V_{1}}⊕F_{V_{2}}⊕F_{V_{3}}⊕F_{V_{4}}.$$

Therefore, the following cases summarise the various binary image classifications implemented:

1. Single Layer: Classification is performed individually using $F_{V_{i}}$, where $V_{i}$ is any of the ocular vascular layers.
2. Two Layers: Classification is performed using $F_{V_{i},V_{j}}$, where $V_{i}$ and $V_{j}$ are any two ocular vascular layers.
3. Three Layers: Classification is performed using $F_{V_{i},V_{j},V_{k}}$, where $V_{i},V_{j},V_{k}$ are any three ocular vascular layers.
4. All Layers: Classification is performed using $F_{global}$, the concatenated feature vector of all ocular vascular layers.

There are several motivations for performing binary image classification in these ways. Analysing each OCTA image from separate ocular vascular layers may help identify the most predictive layer containing information on vascular abnormalities linked to AMD, such as CNV regions. Furthermore, the textural appearance of vascular pathologies related to AMD can be more perceptible in certain ocular vascular layers than in others. Therefore, performing binary image classification by concatenating two feature vectors extracted from two OCTA images of different ocular vascular layers, three feature vectors from three OCTA images, or all feature vectors from all OCTA images may help identify complementary relationships between features from different layers. Additionally, this approach may address the large within-class variation issue and improve the detection of AMD.

However, the Manchester Royal Eye Hospital and Moorfields Eye Hospital OCTA datasets are imbalanced. The number of eyes in the OCTA datasets for each class of eye condition (e.g., healthy, wet AMD, and dry AMD) is unequal. Since identifying all classes of eye conditions is crucial, the following evaluation strategies were conducted on the OCTA image datasets from both hospitals:

1. Employ the stratified K-fold cross-validation strategy with K = 10 to divide the OCTA datasets into 10 stratified folds, ensuring each training and testing set preserves the class distribution (i.e., healthy, wet AMD, and dry AMD). The choice of K = 10 is motivated by empirical results demonstrating this value produces performance estimates that avoid high bias (e.g., overestimated performance) or high variance (e.g., significant fluctuations in performance estimates) [58]. This resampling technique supports reliable evaluation of the proposed algorithms’ predictive performance and mitigates overfitting.
2. Compute the area under the receiver operating characteristic curve (AUC) score to provide equal weight to different eye condition classes in binary classification tasks (e.g., healthy vs. wet AMD, dry AMD vs. wet AMD, and CNV vs. non-CNV).

As the evaluation involved employing the stratified K = 10-fold cross-validation strategy and computing the AUC scores, the mean AUC scores along with the standard deviations (std) were estimated. Hence, the overall performances of the algorithms are estimated based on (mean AUC scores ± std) using the two different machine learning classifiers, specifically the KNN and the SVM.

A hyper-parameter search was conducted for the KNN and SVM classifiers used in the two classification algorithms. This involved defining a grid of hyper-parameter values and evaluating each grid point individually using cross-validation. For the KNN classifier, the hyper-parameters were empirically explored by varying the value of *K* nearest neighbours, k={1,3,5,7,9}, and changing the distance metrics, using Euclidean, Manhattan, and Chebyshev functions.

For the SVM classifier, the penalty parameter *C* was varied with C={0.1,1,5,10,50,100}, alongside different kernel functions: linear, Radial Basis Function (rbf), and Polynomial (poly). When using the rbf and poly kernels, the γ and *d* parameters were fine-tuned, with γ={0.00001,0.0001,0.001,0.01,0.1,1} and d={2,3,4,5,6,7,8,9}. Optimal hyper-parameter combinations for each classifier were selected based on cross-validation to achieve the best classification performance.

Additionally, the parameters of the three texture descriptors, i.e., (p={4,8,12,16} and r={1,2,3,4}) for the $LBP_{p,r}^{2riu}$ and $LBP_{p,r}$, and (S={4,5,6,7} and n={4,8,12,16}) for $BRIEF_{S,n}$, were empirically fine-tuned. The motivation for these evaluations is twofold: firstly, to enable comprehensive evaluation and validation of the proposed $LBP_{p,r}^{2riu}$ descriptor for quantifying AMD textural appearance in OCTA images compared to $LBP_{p,r}$ and $BRIEF_{S,n}$; secondly, to identify optimal ocular vascular-specific parameters for the descriptors, facilitating rich texture representations of AMD in OCTA images.

All experiments were conducted on a personal computer (PC) running Windows 7, equipped with an Intel Core i7 3.4 GHz quad-core processor and 16 GB of RAM. The software environment consisted of Python 2.7, utilising essential libraries such as scikit-learn [59] for machine learning model development and evaluation and OpenCV [60] for image processing. To ensure reproducibility, a fixed random seed of 42 was used across all experiments.

Consequently, the above evaluation strategies provide accurate insight into overall performance and enhance validation for the two developed algorithms. Table 4, Table 5 and Table 6 summarise the best classification results achieved by the automated OCTA image classification algorithm using whole local texture features for the healthy vs. wet AMD, dry AMD vs. wet AMD, and CNV vs. non-CNV tasks, respectively. The optimal components, including the best local texture descriptors and classifiers that improved performance, are also listed.

Table 7, Table 8 and Table 9 summarise the optimal classification results achieved by the automated OCTA image classification algorithm, based on the reduced local texture features proposed for the healthy vs. wet AMD, dry AMD vs. wet AMD, and CNV vs. non-CNV classification tasks, respectively. Additionally, the optimal components, including the best local texture descriptors and classifiers that enhanced performance, are detailed.

## 4. Discussion

This section discusses the significance of classification findings from the evaluation in Section 3. Table 10, Table 11 and Table 12 compare the performance of classification algorithms using reduced local and whole local texture features for the healthy vs. wet AMD, dry AMD vs. wet AMD, and CNV vs. non-CNV tasks, respectively.

Broadly, the classification algorithm based on reduced local texture features achieved the best results in most classification experiments on individual OCTA images of different ocular vascular layers, as shown in Table 10, Table 11 and Table 12. However, performance varied across binary classification tasks. For healthy vs. wet AMD, the reduced local texture algorithm performed best on OCTA images of the superficial inner retina and choriocapillaris layers, while the whole local texture algorithm excelled on deep inner retina images. Both algorithms achieved comparable scores, with mean AUC and std. =0.99±0.00, on outer retina images.

For the dry AMD vs. wet AMD classification task, the algorithm based on reduced local texture features significantly outperformed the one based on whole local texture features in nearly all experiments conducted on individual OCTA images of various ocular vascular layers, except those of the choriocapillaris layer (see Table 11). Conversely, for the CNV vs. non-CNV classification task, the algorithm based on whole local texture features achieved the best performance solely on OCTA images of the superficial inner retina layer. However, the reduced local texture features algorithm demonstrated superior results across almost all other ocular vascular layers.

When performing binary classification tasks based on layer combinations, the algorithm using whole local texture features generally showed superior performance. For instance, perfect classification performance (mean AUC score and std. =1.00±0.00) was achieved by concatenating feature vectors from the OCTA images of the outer retina and choriocapillaris layers for the healthy vs. wet AMD task. However, using reduced local texture features generally yielded better performance for dry AMD vs. wet AMD and CNV vs. non-CNV tasks, achieving mean AUC scores and std. =0.81±0.01 and 0.85±0.05, respectively.

**Figure 9:**
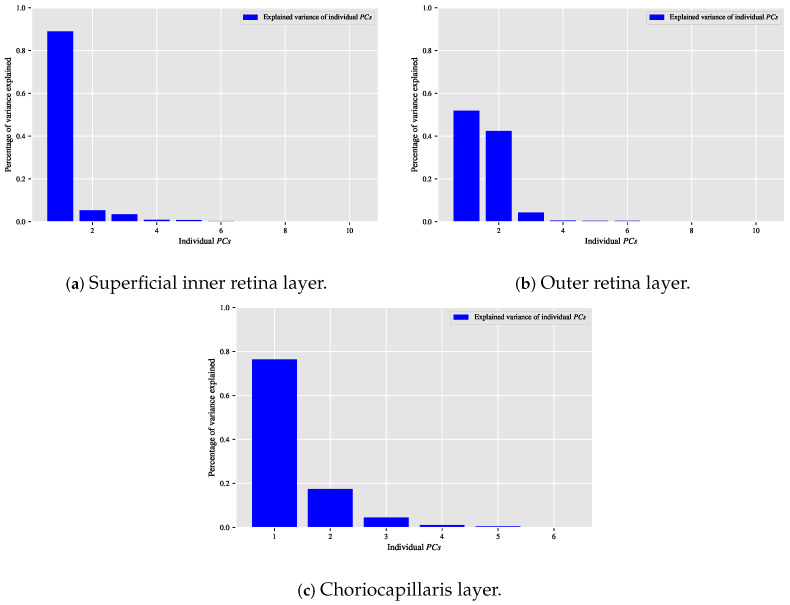
Plots of bar charts that demonstrate the percentage of variance explained by the individual PCs in the healthy vs. wet AMD classification task for only the occasions in which the classification algorithm that is based on reduced local texture features achieved better performance. (**a**) Demonstrates the explained variance ratios of the individual PCs after applying the PCA technique on the feature vectors extracted from the OCTA images of the superficial inner retina layer. (**b**) When applying the PCA technique on the feature vectors extracted from the OCTA images of the outer retina layer. (**c**) When applying the PCA technique on the feature vectors extracted from the OCTA images of the choriocapillaris layer.

**Figure 10:**
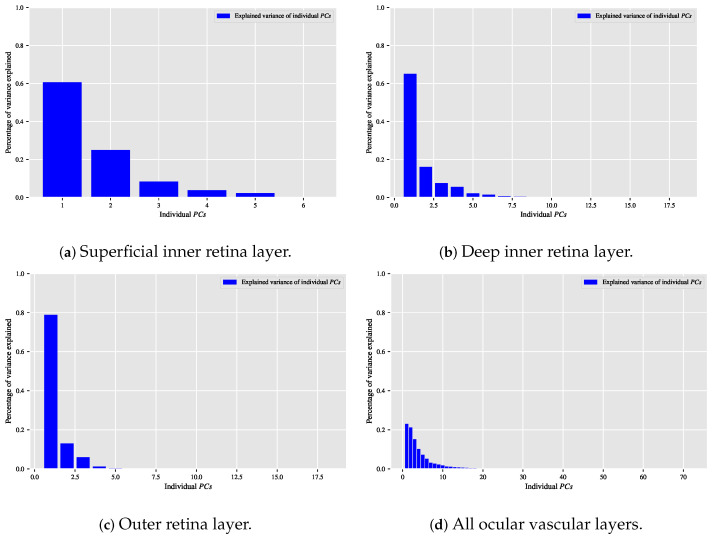
Visualising the percentage of variance explained by the individual PCs in the dry AMD vs. wet AMD classification task for only the occasions in which the classification algorithm that is based on reduced local texture features accomplished improved performance. (**a**) Reveals the explained variance ratios of the individual PCs when applying the PCA technique on the feature vectors extracted from the OCTA images of the superficial inner retina layer. (**b**) When applying the PCA technique on the feature vectors extracted from the OCTA images of the deep inner retina layer. (**c**) When applying the PCA technique on the feature vectors extracted from the OCTA images of the outer retina layer. (**d**) When applying the PCA technique on the combined feature vectors extracted by concatenating the feature vectors of all ocular vascular layers.

**Figure 11:**
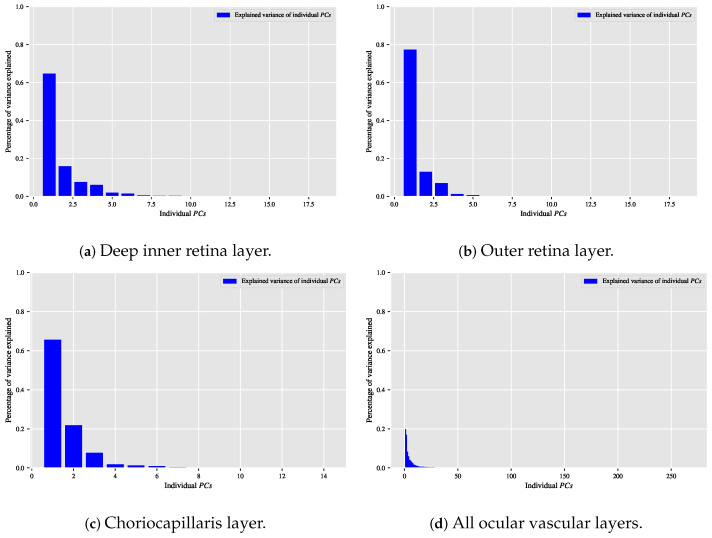
Depicting the percentage of variance explained by the individual PCs in the CNV vs. non-CNV classification task for only the situations in which the classification algorithm that is based on reduced local texture features demonstrated enhanced classification performance. (**a**) Shows the explained variance ratios of the individual PCs when applying the PCA technique on the feature vectors extracted from the OCTA images of the deep inner retina layer. (**b**) When applying the PCA technique on the feature vectors extracted from the OCTA images of the outer retina layer. (**c**) When applying the PCA technique on the feature vectors extracted from the OCTA images of the choriocapillaris layer. (**d**) When applying the PCA technique on the combined feature vectors extracted through concatenating the feature vectors of all ocular vascular layers.

When the classification relationship between local texture features and the individual classes of interest (i.e., dry AMD, wet AMD, and healthy) is influenced by the variability of those features, employing the PCA technique may help establish a suitable relationship between the decorrelated or reduced local texture features and the target classes to be distinguished. Figure 9, Figure 10 and Figure 11 show bar chart plots quantifying the explained variance ratios of individual PCs in binary classification tasks: healthy vs. wet AMD, dry AMD vs. wet AMD, and CNV vs. non-CNV. These plots represent the explained variance ratios of PCs after applying PCA on feature vectors from OCTA images of ocular vascular layers, focusing on cases where reduced local texture features improved classification performance.

Looking at the plots in Figure 9, Figure 10 and Figure 11, the first *K*PCs typically capture most of the variance in the original data. The improved performance of the classification algorithm based on reduced local texture features suggests these problems are tied to the variability in the original features. Retaining 95% of the variance proved effective for image classification tasks while reducing potential redundancies in the original features.

Nevertheless, the classification results achieved by the two purely OCTA data-driven classification algorithms developed in this paper should not be underestimated. Specifically, they recognise subtle texture variations in OCTA images of ocular vascular layers (superficial and deep inner retina) not typically used for AMD detection. These findings are key for diagnosing vascular pathologies related to AMD, e.g., CNV and non-CNV lesions, from OCTA images. Identifying abnormalities in the superficial and deep inner retina layers can greatly aid clinicians, especially when such anomalies are not easily observed in the outer retina and choriocapillaris layers typically used for AMD diagnosis.

Unlike Wang et al. [37], which identifies subjects with wet AMD based solely on visibly perceptible CNV lesions in OCTA images of the outer retina layer, this paper discriminates between healthy subjects and those with various AMD stages (e.g., dry AMD and wet AMD) using OCTA images from different ocular vascular layers, regardless of CNV lesion visibility. This is possible because CNV regions can appear more distinguishable in certain vascular layers than others.

From a wet AMD detection perspective, the optimal binary OCTA image classification task, enabling the most accurate discrimination of wet AMD cases, is the healthy vs. wet AMD task.Table 13 provides an in-depth evaluation of the two classification algorithms developed in this paper for the optimal binary classification task, i.e., healthy vs. wet AMD, which significantly improved wet AMD detection accuracy in OCTA images. These algorithms are assessed using the best components, such as optimal combinations of local texture descriptors and classifiers, as summarised in Table 4 and Table 7. Evaluation results in Table 13 are determined using measures including accuracy, sensitivity (recall), specificity, precision, and AUC, applying stratified K = 10 folds cross-validation. Results are presented as mean scores ± std. for each measure.

While the classification algorithm of Wang et al. [37] achieved perfect sensitivity/recall, i.e., 1.00, it was tested on a single small OCTA dataset manually depth-adjusted to clearly visualise CNV lesion regions. However, its generalisation to unseen OCTA data remains uncertain, as manual depth adjustments are subjective and impractical for routine clinical use. In contrast, the classification algorithms in this paper are evaluated using cross-validation and larger, unaltered OCTA datasets.

The study by Wang et al. [37] demonstrates a sophisticated deep learning approach that combines classification and segmentation tasks through the use of dual CNN architectures. However, their method is heavily reliant on extensive manual pre-processing steps, including depth adjustment of imaging slices, layer subtraction operations, and manual annotation of CNV lesions, all of which introduce potential biases and reduce reproducibility. Furthermore, the segmentation model depends on visual features that are enhanced or even artificially emphasised through these pre-processing procedures. While this design may yield strong performance within the confines of a controlled dataset, it poses significant limitations in terms of practical deployment, particularly in settings where expert annotation and consistent imaging quality cannot be guaranteed.

In contrast, our methods avoid the dependence on complex pre-processing and instead leverages a feature-driven strategy that focuses on the intrinsic textural and vascular characteristics of the OCTA images. The features employed are carefully designed to be robust to variations in imaging quality and to capture diagnostically relevant patterns without requiring intensive manual input or image manipulation. This not only enhances the reproducibility and interpretability of the results but also improves the feasibility of clinical deployment in resource-constrained environments. Additionally, our evaluation is conducted using a statistically rigorous validation framework that enhances the robustness and generalisability of the proposed method.

While both classification algorithms in this paper demonstrated effective results, the algorithm based on reduced local texture features generally outperformed in most binary classification experiments on individual OCTA images of different ocular vascular layers, as shown in Table 10 and Table 13. Conversely, the algorithm using whole local texture features excelled in binary classification experiments conducted via layer combination (see Table 10 and Table 13).

A key contribution of this study lies in the targeted integration of established techniques, specifically LBP descriptors and PCA, within the specific context of OCTA image analysis for AMD classification. Rather than introducing a completely new algorithm, this research focuses on designing an effective, interpretable, and resource-efficient pipeline that can realistically be applied in clinical practice. By performing classification based on the entire OCTA image, we avoid the need for lesion segmentation. This reduces the dependency on detailed manual annotations while directly addressing the challenge of overlapping healthy and pathological textures.

Our findings indicate that reducing the dimensionality of texture features through PCA not only lowers computational requirements but also improves classification accuracy. This improvement is likely due to the removal of inter-class redundancies that often occur in full OCTA images. The results support the importance of domain-informed feature engineering, particularly in medical imaging scenarios where sample sizes are limited and deep learning approaches may not be feasible or easily interpretable.

Although LBP and PCA are not novel techniques on their own, their deliberate and problem-specific adaptation provides a practical contribution. This work demonstrates how existing methods, when carefully tailored to suit the characteristics of medical imaging data, can yield a scalable solution to a complex classification problem.

Furthermore, to evaluate the generalisability of the proposed feature-based frameworks, we employed two classic classification methods: SVM and KNN, which represent distinct decision strategies. These were deliberately chosen for their widespread use in texture-based analysis and their effectiveness in low-data medical imaging contexts. The consistent performance observed across both classifiers highlights the robustness of the extracted features. While this study prioritises feature design over classifier complexity, future work could incorporate additional classification schemes, such as Random Forests or Naïve Bayes, to further assess the adaptability and scalability of the proposed methods.

## 5. Conclusions

To conclude, the diagnostic techniques developed in this paper have effectively achieved their intended objectives. However, there are several opportunities for further refinement, comprehensive evaluation, and wider application. These opportunities are summarised as follows:

The automated diagnostic classification algorithms demonstrated excellent performance in differentiating healthy subjects from wet AMD patients across various OCTA images. Consequently, extending these algorithms to distinguish healthy individuals from patients with other ocular disorders, such as diabetic retinopathy (DR) or glaucoma, would be a valuable direction. Achieving this will require the acquisition of carefully curated OCTA image data representing DR or glaucoma conditions.

Evaluating automated diagnostic classification algorithms on more complex tasks offers significant potential. For example, it is clinically valuable to quantify and differentiate variations among wet AMD patients, such as distinguishing between those with active CNV lesions requiring treatment and those with inactive CNV lesions suitable for observation. Achieving this will require carefully curated OCTA image data representing these wet AMD variations. Additionally, the OCTA image datasets used in this research are smaller than ideal. Collecting a much larger dataset would enable comprehensive assessments of the automated diagnostic algorithms developed in this study, ensuring robust performance evaluation and validation.

Finally, the trend towards automated image texture analysis for medical image diagnostics, such as classification or segmentation, emphasises deeper, more complex architectures. Exploring deep CNN models within this context is promising but requires curated OCTA datasets representing various eye conditions, including healthy eyes and AMD. Notably, data augmentation techniques, while increasing dataset size, are typically unsuitable here as they may distort OCTA textures, leading to misleading results. In addition, future research may benefit from investigating novel deep learning techniques, such as capsule networks with residual pose routing [61], which have shown promise in capturing spatial hierarchies and part-whole relationships that could enhance the model’s ability to interpret complex OCTA textures.

## Figures and Tables

**Table 1:** A summary of the multiclass image classification results of the various CNN models proposed by Vaghefi et al. [36].

Design of CNN Model	Evaluation Measures	Age and Eye Condition		
		YH	OH	Dry AMD
CNN Model based on OCTA	Sensitivity/recall	95%	83%	97%
	Specificity	99%	96%	76%
	Accuracy	91%		
CNN Model based on OCTA + OCT	Sensitivity/recall	100%	95%	92%
	Specificity	97%	94%	98%
	Accuracy	96%		
CNN Model based on OCTA + OCT + CFP	Sensitivity/recall	100%	99%	100%
	Specificity	100%	100%	99%
	Accuracy	99%		

**Table 2:** Summary of the total number of images in the OCTA image dataset provided by the Manchester Royal Eye Hospital.

Subjects	Ocular Vascular Tissue Layers				Total
	Superficial Inner Retina	Deep Inner Retina	Outer Retina	Choriocapillaris	
Healthy	33	33	33	33	132
Wet AMD	23	23	23	23	92

**Table 3:** Summary of the total number of images in the OCTA image dataset provided by the Moorfields Eye Hospital.

Subjects	Ocular Vascular Tissue Layers				Total
	Superficial Inner Retina	Deep Inner Retina	Outer Retina	Choriocapillaris	
Wet AMD	166	166	166	166	664
Dry AMD	79	79	79	79	316
Secondary CNV	25	25	25	25	100

**Table 4:** A summary of best classification results (mean AUC scores ± std.) as achieved by the automated OCTA image classification algorithm proposed for solving the healthy vs. wet AMD classification task based on whole local texture features.

Ocular Vascular Layers	Optimal Descriptors	Optimal Classifiers	AUC ± std
Superficial inner retina layer	$LBP_{4,1}^{2riu}$	KNN ($D_{euclidean}^{k=3}$)	0.89±0.05
Deep inner retina layer	$LBP_{16,4}^{2riu}$	KNN ($D_{euclidean}^{k=3}$)	0.96±0.04
Outer retina layer	$LBP_{all\;values}^{2riu}$ / $LBP_{all\;values}$	KNN ($D_{euclidean}^{k=3}$) / SVM ($K_{linear}^{C=1}$)	0.99±0.00
Choriocapillaris layer	$LBP_{16,4}^{2riu}$	SVM ($K_{linear}^{C=10}$)	0.97±0.03
Superficial + Deep	$LBP_{4,1}^{2riu}$	SVM ($K_{linear}^{C=1}$)	0.94±0.03
Superficial + Outer	$LBP_{16,4}^{2riu}$	KNN ($D_{euclidean}^{k=1}$)	0.98±0.02
Superficial + Choriocapillaris	$LBP_{12,3}$	KNN ($D_{manhattan}^{k=3}$)	0.94±0.04
Deep + Outer	$LBP_{16,4}^{2riu}$	KNN ($D_{euclidean}^{k=1}$)	0.98±0.01
Deep + Choriocapillaris	$LBP_{16,4}$	KNN ($D_{manhattan}^{k=5}$)	0.98±0.01
Outer + Choriocapillaris	$LBP_{16,4}^{2riu}$	KNN ($D_{euclidean}^{k=3}$)	**1.00 ± 0.00**
Superficial + Deep + Outer	$LBP_{16,4}^{2riu}$	KNN ($D_{euclidean}^{k=3}$)	0.98±0.01
Superficial + Deep + Choriocapillaris	$LBP_{16,4}$	KNN ($D_{manhattan}^{k=5}$)	0.98±0.02
Superficial + Outer + Choriocapillaris	$LBP_{16,4}^{2riu}$	KNN ($\;D_{euclidean}^{k=3}$)	0.97±0.02
Deep + Outer + Choriocapillaris	$LBP_{16,4}^{2riu}$	KNN ($D_{euclidean}^{k=3}$)	0.98±0.03
All ocular vascular layers	$LBP_{12,3}^{2riu}$	KNN ($D_{euclidean}^{k=3}$)	0.99±0.03

**Table 5:** A summary of best classification results (mean AUC scores ± std.) as achieved by the automated OCTA image classification algorithm proposed for solving the dry AMD vs. wet AMD classification task based on whole local texture features.

Ocular Vascular Layers	Optimal Descriptors	Optimal Classifiers	AUC ± std
Superficial inner retina layer	$LBP_{4,1}^{2riu}$	SVM ($K_{linear}^{C=1}$)	0.71±0.06
Deep inner retina layer	$LBP_{16,4}^{2riu}$	SVM ($K_{linear}^{C=1}$)	0.75±0.04
Outer retina layer	$LBP_{16,4}^{2riu}$	SVM ($K_{linear}^{C=1}$)	0.83±0.03
Choriocapillaris layer	$LBP_{12,3}^{2riu}$	SVM ($K_{rbf}^{C=50,\gamma =0.1}$)	0.83±0.01
Superficial + Deep	$LBP_{4,1}^{2riu}$	SVM ($K_{poly}^{C=1,d=2}$)	0.71±0.09
Superficial + Outer	$LBP_{12,3}$	KNN ($D_{manhattan}^{k=3}$)	0.74±0.05
Superficial + Choriocapillaris	$LBP_{4,1}^{2riu}$	SVM ($K_{linear}^{C=10}$)	0.76±0.05
Deep + Outer	$LBP_{8,2}^{2riu}$	SVM ($K_{poly}^{C=1,d=3}$)	0.75±0.05
Deep + Choriocapillaris	$LBP_{12,3}^{2riu}$	SVM ($K_{poly}^{C=1,d=3}$)	0.78±0.06
Outer + Choriocapillaris	$LBP_{12,3}^{2riu}$	SVM ($K_{poly}^{C=10,d=3}$)	0.77±0.06
Superficial + Deep + Outer	$LBP_{8,2}^{2riu}$	SVM ($K_{poly}^{C=10,d=3}$)	0.79±0.07
Superficial + Deep + Choriocapillaris	$LBP_{4,1}^{2riu}$	SVM ($K_{poly}^{C=10,d=3}$)	0.79±0.06
Superficial + Outer + Choriocapillaris	$LBP_{8,2}$	SVM ($K_{poly}^{C=10,d=3}$)	0.78±0.05
Deep + Outer + Choriocapillaris	$LBP_{8,2}^{2riu}$	SVM ($K_{poly}^{C=10,d=3}$)	0.80±0.06
All ocular vascular layers	$LBP_{8,2}^{2riu}$	SVM ($K_{linear}^{C=100}$)	0.79±0.04

**Table 6:** A summary of best classification results (mean AUC scores ± std.) as achieved by the automated OCTA image classification algorithm proposed for solving the CNV vs. non-CNV classification task based on whole local texture features.

Ocular Vascular Layers	Optimal Descriptors	Optimal Classifiers	AUC ± std
Superficial inner retina layer	$LBP_{8,2}^{2riu}$	SVM ($K_{linear}^{C=1}$)	0.70±0.05
Deep inner retina layer	$LBP_{12,3}^{2riu}$	SVM ($K_{linear}^{C=1}$)	0.72±0.03
Outer retina layer	$LBP_{16,4}^{2riu}$	SVM ($K_{linear}^{C=1}$)	0.82±0.03
Choriocapillaris layer	$LBP_{16,4}^{2riu}$	SVM ($K_{linear}^{C=1}$)	0.81±0.04
Superficial + Deep	$LBP_{4,1}^{2riu}$	SVM ($K_{poly}^{C=10,d=3}$)	0.68±0.04
Superficial + Outer	$LBP_{8,2}^{2riu}$	SVM ($K_{poly}^{C=10,d=3}$)	0.79±0.06
Superficial + Choriocapillaris	$LBP_{8,2}^{2riu}$	SVM ($K_{poly}^{C=10,d=3}$)	0.75±0.06
Deep + Outer	$LBP_{8,2}^{2riu}$	SVM ($K_{poly}^{C=10,d=3}$)	0.79±0.07
Deep + Choriocapillaris	$LBP_{12,3}$	KNN ($D_{manhattan}^{k=5}$)	0.76±0.04
Outer + Choriocapillaris	$LBP_{8,2}^{2riu}$	SVM ($K_{poly}^{C=10,d=3}$)	0.82±0.04
Superficial + Deep + Outer	$LBP_{8,2}^{2riu}$	SVM ($K_{poly}^{C=10,d=3}$)	0.80±0.07
Superficial + Deep + Choriocapillaris	$LBP_{8,2}^{2riu}$	SVM ($K_{poly}^{C=10,d=3}$)	0.77±0.05
Superficial + Outer + Choriocapillaris	$LBP_{12,3}^{2riu}$	KNN ($D_{manhattan}^{k=5}$)	0.82±0.04
Deep + Outer + Choriocapillaris	$LBP_{12,3}$	KNN ($D_{manhattan}^{k=5}$)	0.82±0.03
All ocular vascular layers	$LBP_{12,3}$	KNN ($D_{manhattan}^{k=7}$)	0.81±0.03

**Table 7:** A summary of best classification results (mean AUC scores ± std.) as achieved by the automated OCTA image classification algorithm proposed for solving the healthy vs. wet AMD classification task based on reduced local texture features.

Ocular Vascular Layers	Optimal Descriptors	Optimal Classifiers	AUC ± std
Superficial inner retina layer	$LBP_{8,2}^{2riuPCA}$	KNN ($D_{manhattan}^{k=7}$)	0.90±0.02
Deep inner retina layer	$LBP_{16,4}^{2riuPCA}$	SVM ($K_{linear}^{C=10}$)	0.95±0.03
Outer retina layer	$LBP_{p=4,8,r=1,2}^{2riuPCA}$ / $LBP_{all\;values}^{PCA}$	KNN ($D_{euclidean,manhattan}^{k=3,9}$) / SVM ($K_{linear}^{C=1,10}$)	0.99±0.00
Choriocapillaris layer	$LBP_{4,1}^{2riuPCA}$	KNN ($D_{euclidean}^{k=3}$) / SVM ($K_{linear}^{C=1}$)	0.99±0.00
Superficial + Deep	$LBP_{16,4}^{2riuPCA}$	SVM ($K_{linear}^{C=10}$)	0.93±0.05
Superficial + Outer	$LBP_{12,3}^{PCA}$	KNN ($D_{euclidean}^{k=1}$)	0.95±0.04
Superficial + Choriocapillaris	$LBP_{12,3}^{PCA}$	KNN ($D_{manhattan}^{k=3}$)	0.92±0.07
Deep + Outer	$LBP_{12,3}^{PCA}$	KNN ($D_{euclidean}^{k=1}$)	0.95±0.03
Deep + Choriocapillaris	$LBP_{16,4}^{2riuPCA}$	SVM ($K_{rbf}^{C=1,\gamma =0.1}$)	0.94±0.03
Outer + Choriocapillaris	$LBP_{16,4}^{2riuPCA}$	KNN ($D_{euclidean}^{k=5}$)	0.98±0.03
Superficial + Deep + Outer	$LBP_{16,4}^{PCA}$	KNN ($D_{euclidean}^{k=1}$)	0.96±0.04
Superficial + Deep + Choriocapillaris	$LBP_{16,4}^{2riuPCA}$	KNN ($D_{manhattan}^{k=7}$)	0.97±0.05
Superficial + Outer + Choriocapillaris	$LBP_{16,4}^{2riuPCA}$	KNN ($D_{euclidean}^{k=5}$)	0.97±0.03
Deep + Outer + Choriocapillaris	$LBP_{16,4}^{PCA}$	KNN ($D_{euclidean}^{k=1}$)	0.97±0.02
All ocular vascular layers	$LBP_{16,4}^{2riuPCA}$	KNN ($D_{manhattan}^{k=3}$) / SVM ($K_{linear}^{C=1}$)	0.98±0.01

**Table 8:** A summary of best classification results (mean AUC scores ± std.) as achieved by the automated OCTA image classification algorithm proposed for solving the dry AMD vs. wet AMD classification task based on reduced local texture features.

Ocular Vascular Layers	Optimal Descriptors	Optimal Classifiers	AUC ± std
Superficial inner retina layer	$LBP_{4,1}^{2riuPCA}$	SVM ($K_{linear}^{C=1}$)	0.74±0.04
Deep inner retina layer	$LBP_{16,4}^{2riuPCA}$	SVM ($K_{rbf}^{C=1,\gamma =0.01}$)	0.79±0.03
Outer retina layer	$LBP_{16,4}^{2riuPCA}$	SVM ($K_{linear}^{C=50}$)	0.85±0.02
Choriocapillaris layer	$LBP_{12,3}^{2riuPCA}$	SVM($K_{linear}^{C=1}$)	0.83±0.02
Superficial + Deep	$LBP_{4,1}^{PCA}$	SVM ($K_{poly}^{C=10,d=3}$)	0.65±0.05
Superficial + Outer	$LBP_{16,4}^{PCA}$	KNN ($D_{manhattan}^{k=3}$)	0.66±0.03
Superficial + Choriocapillaris	$LBP_{16,4}^{2riuPCA}$	KNN ($D_{euclidean}^{k=3}$)	0.67±0.03
Deep + Outer	$LBP_{8,2}^{PCA}$	SVM ($K_{poly}^{C=10,d=3}$)	0.67±0.04
Deep + Choriocapillaris	$LBP_{16,4}^{PCA}$	SVM ($K_{rbf}^{C=1,\gamma =0.1}$)	0.69±0.04
Outer + Choriocapillaris	$LBP_{12,3}^{2riuPCA}$	SVM ($K_{rbf}^{C=10,\gamma =0.01}$)	0.74±0.07
Superficial + Deep + Outer	$LBP_{8,2}^{PCA}$	SVM ($K_{poly}^{C=10,d=3}$)	0.70±0.04
Superficial + Deep + Choriocapillaris	$LBP_{8,2}^{PCA}$	KNN ($D_{euclidean}^{k=5}$)	0.77±0.05
Superficial + Outer + Choriocapillaris	$LBP_{12,3}^{PCA}$	SVM ($K_{poly}^{C=10,d=3}$)	0.77±0.05
Deep + Outer + Choriocapillaris	$LBP_{12,3}^{2riuPCA}$	SVM ($K_{rbf}^{C=10,\gamma =0.01}$)	0.79±0.05
All ocular vascular layers	$LBP_{16,4}^{2riuPCA}$	SVM ($K_{rbf}^{C=10,\gamma =0.001}$)	0.81±0.01

**Table 9:** A summary of best classification results (mean AUC scores ± std.) as achieved by the automated OCTA image classification algorithm proposed for solving the CNV vs. non-CNV classification task based on reduced local texture features.

Ocular Vascular Layers	Optimal Descriptors	Optimal Classifiers	AUC ± std
Superficial inner retina layer	$LBP_{8,2}^{2riuPCA}$	SVM ($K_{rbf}^{C=1,\gamma =0.1}$)	0.66±0.01
Deep inner retina layer	$LBP_{16,4}^{2riuPCA}$	SVM ($K_{poly}^{C=10,d=3}$)	0.76±0.03
Outer retina layer	$LBP_{16,4}^{2riuPCA}$	SVM ($K_{linear}^{C=1}$)	0.83±0.03
Choriocapillaris layer	$LBP_{12,3}^{2riuPCA}$	SVM ($K_{rbf}^{C=10,\gamma =0.001}$)	0.81±0.02
Superficial + Deep	$LBP_{16,4}^{2riuPCA}$	KNN ($D_{manhattan}^{k=3}$)	0.65±0.05
Superficial + Outer	$LBP_{12,3}^{PCA}$	SVM ($K_{linear}^{C=10}$)	0.68±0.05
Superficial + Choriocapillaris	$LBP_{8,2}^{PCA}$	KNN ($D_{manhattan}^{k=5}$)	0.68±0.05
Deep + Outer	$LBP_{8,2}^{2riuPCA}$	SVM ($K_{linear}^{C=10}$)	0.69±0.04
Deep + Choriocapillaris	$LBP_{12,3}^{PCA}$	KNN ($D_{manhattan}^{k=5}$)	0.70±0.04
Outer + Choriocapillaris	$LBP_{12,3}^{2riuPCA}$	SVM ($K_{rbf}^{C=10,\gamma =0.01}$)	0.79±0.03
Superficial + Deep + Outer	$LBP_{12,3}^{2riuPCA}$	SVM ($K_{linear}^{C=1}$)	0.77±0.05
Superficial + Deep + Choriocapillaris	$LBP_{8,2}^{2riuPCA}$	SVM ($K_{linear}^{C=10}$)	0.74±0.05
Superficial + Outer + Choriocapillaris	$LBP_{12,3}^{PCA}$	SVM ($K_{poly}^{C=10,d=3}$)	0.79±0.04
Deep + Outer + Choriocapillaris	$LBP_{12,3}^{2riuPCA}$	SVM ($K_{rbf}^{C=10,\gamma =0.01}$)	0.80±0.05
All ocular vascular layers	$LBP_{8,2}^{PCA}$	SVM ($K_{rbf}^{C=10,\gamma =0.001}$)	0.85±0.05

**Table 10:** Performance comparison based on mean AUC scores ± std. of the two automated OCTA image classification algorithms proposed for solving the healthy vs. wet AMD classification task based on whole local texture features and reduced local texture features.

Ocular Vascular Layers	Whole Local Features	Reduced Local Features
Superficial inner retina layer	0.89±0.05	0.90±0.02
Deep inner retina layer	0.96±0.04	0.95±0.03
Outer retina layer	0.99±0.00	0.99±0.00
Choriocapillaris layer	0.97±0.03	0.99±0.00
Superficial + Deep	0.94±0.03	0.93±0.05
Superficial + Outer	0.98±0.02	0.95±0.04
Superficial + Choriocapillaris	0.94±0.04	0.92±0.07
Deep + Outer	0.98±0.01	0.95±0.03
Deep + Choriocapillaris	0.98±0.01	0.94±0.03
Outer + Choriocapillaris	1.00±0.00	0.98±0.03
Superficial + Deep + Outer	0.98±0.01	0.96±0.04
Superficial + Deep + Choriocapillaris	0.98±0.02	0.97±0.05
Superficial + Outer + Choriocapillaris	0.97±0.02	0.97±0.03
Deep + Outer + Choriocapillaris	0.98±0.03	0.97±0.02
All ocular vascular layers	0.99±0.03	0.98±0.01

**Table 11:** Performance comparison based on mean AUC scores ± std. of the two automated OCTA image classification algorithms proposed for solving the dry AMD vs. wet AMD classification task based on whole local texture features and reduced local texture features.

Ocular Vascular Layers	Whole Local Features	Reduced Local Features
Superficial inner retina layer	0.71±0.06	0.74±0.04
Deep inner retina layer	0.75±0.04	0.79±0.03
Outer retina layer	0.83±0.03	0.85±0.02
Choriocapillaris layer	0.83±0.01	0.83±0.02
Superficial + Deep	0.71±0.09	0.65±0.05
Superficial + Outer	0.74±0.05	0.66±0.03
Superficial + Choriocapillaris	0.76±0.05	0.67±0.03
Deep + Outer	0.75±0.05	0.67±0.04
Deep + Choriocapillaris	0.78±0.06	0.69±0.04
Outer + Choriocapillaris	0.77±0.06	0.74±0.07
Superficial + Deep + Outer	0.79±0.07	0.70±0.04
Superficial + Deep + Choriocapillaris	0.79±0.06	0.77±0.05
Superficial + Outer + Choriocapillaris	0.78±0.05	0.77±0.05
Deep + Outer + Choriocapillaris	0.80±0.06	0.79±0.05
All ocular vascular layers	0.79±0.04	0.81±0.01

**Table 12:** Performance comparison based on mean AUC scores ± std. of the two automated OCTA image classification algorithms proposed for solving the CNV vs. non-CNV classification task based on whole local texture features and reduced local texture features.

Ocular Vascular Layers	Whole Local Features	Reduced Local Features
Superficial inner retina layer	0.70±0.05	0.66±0.01
Deep inner retina layer	0.72±0.03	0.76±0.03
Outer retina layer	0.82±0.03	0.83±0.03
Choriocapillaris layer	0.81±0.04	0.81±0.02
Superficial + Deep	0.68±0.04	0.65±0.05
Superficial + Outer	0.79±0.06	0.68±0.05
Superficial + Choriocapillaris	0.75±0.06	0.68±0.05
Deep + Outer	0.79±0.07	0.69±0.04
Deep + Choriocapillaris	0.76±0.04	0.70±0.04
Outer + Choriocapillaris	0.82±0.04	0.79±0.03
Superficial + Deep + Outer	0.80±0.07	0.77±0.05
Superficial + Deep + Choriocapillaris	0.77±0.05	0.74±0.05
Superficial + Outer + Choriocapillaris	0.82±0.04	0.79±0.04
Deep + Outer + Choriocapillaris	0.82±0.03	0.80±0.05
All ocular vascular layers	0.81±0.03	0.85±0.05

**Table 13:** An in-depth evaluation of the two automated OCTA image classification algorithms proposed that are based on whole local texture features and reduced local texture features on the optimal binary classification task, i.e., healthy vs. wet AMD.

Ocular Vascular Layers	Evaluation Measures	Whole Local Features	Reduced Local Features
	AUC	0.89±0.05	**0.90 ± 0.02**
	Accuracy	0.82±0.05	0.83±0.05
Superficial Inner Retina Layer	Sensitivity/Recall	1.00±0.00	0.78±0.10
	Specificity	0.83±0.09	0.85±0.10
	Precision	0.90±0.07	0.92±0.06
	AUC	0.96±0.04	0.95±0.03
	Accuracy	0.91±0.05	0.88±0.05
Deep Inner Retina Layer	Sensitivity/Recall	0.84±0.10	0.90±0.10
	Specificity	0.95±0.06	0.90±0.10
	Precision	1.00±0.00	0.90±0.07
	AUC	0.99±0.00	0.99±0.00
	Accuracy	0.98±0.02	1.00±0.00
Outer Retina Layer	Sensitivity/Recall	1.00±0.00	1.00±0.00
	Specificity	0.96±0.05	0.99±0.00
	Precision	0.97±0.03	1.00±0.00
	AUC	0.97±0.03	0.99±0.00
	Accuracy	0.94±0.03	0.97±0.03
Choriocapillaris layer	Sensitivity/Recall	1.00±0.00	1.00±0.00
	Specificity	0.88±0.06	0.92±0.06
	Precision	0.92±0.05	0.96±0.05
	AUC	0.94±0.03	0.93±0.05
	Accuracy	0.86±0.08	0.85±0.07
Superficial + Deep	Sensitivity/Recall	0.80±0.09	0.85±0.10
	Specificity	0.93±0.06	0.82±0.11
	Precision	0.95±0.06	0.90±0.07
	AUC	0.98±0.02	0.95±0.04
	Accuracy	0.94±0.05	0.93±0.04
Superficial + Outer	Sensitivity/Recall	0.98±0.04	0.96±0.04
	Specificity	0.89±0.09	0.93±0.08
	Precision	0.92±0.06	0.96±0.05
	AUC	0.94±0.04	0.92±0.07
	Accuracy	0.85±0.06	0.82±0.10
Superficial + Choriocapillaris	Sensitivity/Recall	0.89±0.08	0.92±0.06
	Specificity	0.91±0.08	0.84±0.09
	Precision	0.90±0.06	0.81±0.08
	AUC	0.98±0.01	0.95±0.03
	Accuracy	0.98±0.03	0.93±0.05
Deep + Outer	Sensitivity/Recall	1.00±0.00	0.95±0.07
	Specificity	0.96±0.04	0.94±0.08
	Precision	0.98±0.03	0.93±0.05
	AUC	0.98±0.01	0.94±0.03
	Accuracy	0.92±0.04	0.90±0.06
Deep + Choriocapillaris	Sensitivity/Recall	0.91±0.08	0.90±0.09
	Specificity	0.95±0.06	0.95±0.08
	Precision	0.96±0.05	0.93±0.04
	AUC	1.00±0.00	0.98±0.03
	Accuracy	1.00±0.00	0.97±0.03
Outer + Choriocapillaris	Sensitivity/Recall	1.00±0.00	0.95±0.04
	Specificity	1.00±0.00	1.00±0.00
	Precision	1.00±0.00	1.00±0.00
	AUC	0.98±0.01	0.96±0.04
	Accuracy	0.97±0.03	0.93±0.03
Superficial + Deep + Outer	Sensitivity/Recall	0.97±0.04	0.98±0.04
	Specificity	1.00±0.00	0.80±0.10
	Precision	0.98±0.03	0.91±0.06
	AUC	0.98±0.02	0.97±0.05
	Accuracy	0.94±0.05	0.90±0.02
Superficial + Deep + Choriocapillaris	Sensitivity/Recall	0.96±0.04	0.90±0.06
	Specificity	0.90±0.06	0.93±0.07
	Precision	0.94±0.06	0.92±0.06
	AUC	0.97±0.02	0.97±0.03
	Accuracy	0.97±0.04	0.92±0.05
Superficial + Outer + Choriocapillaris	Sensitivity/Recall	0.97±0.04	0.89±0.09
	Specificity	0.96±0.06	0.97±0.05
	Precision	0.98±0.03	0.97±0.04
	AUC	0.98±0.03	0.97±0.02
	Accuracy	0.95±0.05	0.97±0.04
Deep + Outer + Choriocapillaris	Sensitivity/Recall	0.92±0.09	0.97±0.07
	Specificity	1.00±0.00	0.96±0.04
	Precision	1.00±0.00	0.96±0.07
	AUC	0.99±0.03	0.98±0.01
	Accuracy	0.99±0.02	0.97±0.03
All ocular vascular layers	Sensitivity/Recall	0.98±0.04	0.96±0.04
	Specificity	1.00±0.00	1.00±0.00
	Precision	1.00±0.00	0.99±0.03

## Data Availability

The datasets analysed in this study are not publicly available due to privacy and ethical restrictions. However, they are available from the corresponding author upon reasonable request.

## References

[1] Colijn J.M., Buitendijk G.H., Prokofyeva E., Alves D., Cachulo M.L., Khawaja A.P., Cougnard-Gregoire A., Merle B.M., Korb C., Erke M.G. (2017). Prevalence of age-related macular degeneration in Europe: The past and the future. Ophthalmology.

[2] Bourne R.R., Jonas J.B., Flaxman S.R., Keeffe J., Leasher J., Naidoo K., Parodi M.B., Pesudovs K., Price H., White R.A. (2014). Prevalence and causes of vision loss in high-income countries and in Eastern and Central Europe: 1990–2010. Br. J. Ophthalmol..

[3] Mitchell P., Liew G., Gopinath B., Wong T.Y. (2018). Age-related macular degeneration. Lancet.

[4] Mehta H., Tufail A., Daien V., Lee A.Y., Nguyen V., Ozturk M., Barthelmes D., Gillies M.C. (2018). Real-world outcomes in patients with neovascular age-related macular degeneration treated with intravitreal vascular endothelial growth factor inhibitors. Prog. Retin. Eye Res..

[5] Jia Y., Bailey S.T., Hwang T.S., McClintic S.M., Gao S.S., Pennesi M.E., Flaxel C.J., Lauer A.K., Wilson D.J., Hornegger J. (2015). Quantitative optical coherence tomography angiography of vascular abnormalities in the living human eye. Proc. Natl. Acad. Sci. USA.

[6] Jia Y., Bailey S.T., Wilson D.J., Tan O., Klein M.L., Flaxel C.J., Potsaid B., Liu J.J., Lu C.D., Kraus M.F. (2014). Quantitative optical coherence tomography angiography of choroidal neovascularization in age-related macular degeneration. Ophthalmology.

[7] De Carlo T.E., Romano A., Waheed N.K., Duker J.S. (2015). A review of optical coherence tomography angiography (OCTA). Int. J. Retin. Vitr..

[8] Kuehlewein L., Bansal M., Lenis T.L., Iafe N.A., Sadda S.R., Bonini Filho M.A., Talisa E., Waheed N.K., Duker J.S., Sarraf D. (2015). Optical coherence tomography angiography of type 1 neovascularization in age-related macular degeneration. Am. J. Ophthalmol..

[9] Sulzbacher F., Pollreisz A., Kaider A., Kickinger S., Sacu S., Schmidt-Erfurth U., Center V.E.S. (2017). Identification and clinical role of choroidal neovascularization characteristics based on optical coherence tomography angiography. Acta Ophthalmol..

[10] Liu L., Gao S.S., Bailey S.T., Huang D., Li D., Jia Y. (2015). Automated choroidal neovascularization detection algorithm for optical coherence tomography angiography. Biomed. Opt. Express.

[11] Spaide R.F., Fujimoto J.G., Waheed N.K., Sadda S.R., Staurenghi G. (2018). Optical coherence tomography angiography. Prog. Retin. Eye Res..

[12] de Carlo T.E., Bonini Filho M.A., Chin A.T., Adhi M., Ferrara D., Baumal C.R., Witkin A.J., Reichel E., Duker J.S., Waheed N.K. (2015). Spectral-Domain Optical Coherence Tomography Angiography of Choroidal Neovascularization. Ophthalmology.

[13] Hormel T.T., Hwang T.S., Bailey S.T., Wilson D.J., Huang D., Jia Y. (2021). Artificial intelligence in OCT angiography. Prog. Retin. Eye Res..

[14] Kashani A.H., Chen C.L., Gahm J.K., Zheng F., Richter G.M., Rosenfeld P.J., Shi Y., Wang R.K. (2017). Optical coherence tomography angiography: A comprehensive review of current methods and clinical applications. Prog. Retin. Eye Res..

[15] Simon P., Uma V. (2018). Review of texture descriptors for texture classification. Data Engineering and Intelligent Computing.

[16] Depeursinge A., Fageot J., Al-Kadi O.S., Depeursinge A., Al-Kadi O.S., Mitchell J. (2017). Chapter 1—Fundamentals of Texture Processing for Biomedical Image Analysis: A General Definition and Problem Formulation. Biomedical Texture Analysis.

[17] Coscas G.J., Lupidi M., Coscas F., Cagini C., Souied E.H. (2015). Optical coherence tomography angiography versus traditional multimodal imaging in assessing the activity of exudative age-related macular degeneration: A new diagnostic challenge. Retina.

[18] El Ameen A., Cohen S.Y., Semoun O., Miere A., Srour M., Quaranta-El Maftouhi M., Oubraham H., Blanco-Garavito R., Querques G., Souied E.H. (2015). Type 2 neovascularization secondary to age-related macular degeneration imaged by optical coherence tomography angiography. Retina.

[19] Miere A., Querques G., Semoun O., Capuano V., Souied E.H. (2015). Optical coherence tomography angiography in early type 3 neovascularization. Retina.

[20] Miere A., Querques G., Semoun O., Amoroso F., Zambrowski O., Chapron T., Capuano V., Souied E.H. (2017). Optical coherence tomography angiography changes in early type 3 neovascularization after anti-vascular endothelial growth factor treatment. Retina.

[21] Choi M., Ahn S., Yun C., Kim S.W. (2021). Quantitative OCT angiography findings according to pattern classification of type 1 neovascularization exudative age-related macular degeneration. Eye.

[22] Lupidi M., Cerquaglia A., Chhablani J., Fiore T., Singh S.R., Cardillo Piccolino F., Corbucci R., Coscas F., Coscas G., Cagini C. (2018). Optical coherence tomography angiography in age-related macular degeneration: The game changer. Eur. J. Ophthalmol..

[23] Robinson P.J. (1997). Radiology’s Achilles’ heel: Error and variation in the interpretation of the Röntgen image. Br. J. Radiol..

[24] Tourassi G., Voisin S., Paquit V., Krupinski E. (2013). Investigating the link between radiologists’ gaze, diagnostic decision, and image content. J. Am. Med. Inform. Assoc..

[25] Aziz Z.A., Wells A.U., Hansell D.M., Bain G.A., Copley S.J., Desai S.R., Ellis S.M., Gleeson F.V., Grubnic S., Nicholson A.G. (2004). HRCT diagnosis of diffuse parenchymal lung disease: Inter-observer variation. Thorax.

[26] Tourassi G.D. (1999). Journey toward computer-aided diagnosis: Role of image texture analysis. Radiology.

[27] Ojala T., Pietikainen M., Maenpaa T. (2002). Multiresolution gray-scale and rotation invariant texture classification with local binary patterns. IEEE Trans. Pattern Anal. Mach. Intell..

[28] Coscas G., Lupidi M., Coscas F., Français C., Cagini C., Souied E.H. (2015). Optical coherence tomography angiography during follow-up: Qualitative and quantitative analysis of mixed type I and II choroidal neovascularization after vascular endothelial growth factor trap therapy. Ophthalmic Res..

[29] Muakkassa N.W., Chin A.T., De Carlo T., Klein K.A., Baumal C.R., Witkin A.J., Duker J.S., Waheed N.K. (2015). Characterizing the effect of anti-vascular endothelial growth factor therapy on treatment-naive choroidal neovascularization using optical coherence tomography angiography. Retina.

[30] Palejwala N.V., Jia Y., Gao S.S., Liu L., Flaxel C.J., Hwang T.S., Lauer A.K., Wilson D.J., Huang D., Bailey S.T. (2015). Detection of non-exudative choroidal neovascularization in age-related macular degeneration with optical coherence tomography angiography. Retina.

[31] Yao X., Alam M.N., Le D., Toslak D. (2020). Quantitative optical coherence tomography angiography: A review. Exp. Biol. Med..

[32] Kuehlewein L., Sadda S., Sarraf D. (2015). OCT angiography and sequential quantitative analysis of type 2 neovascularization after ranibizumab therapy. Eye.

[33] Al-Sheikh M., Iafe N.A., Phasukkijwatana N., Sadda S.R., Sarraf D. (2018). Biomarkers of neovascular activity in age-related macular degeneration using optical coherence tomography angiography. Retina.

[34] Zhang Q., Chen C.L., Chu Z., Zheng F., Miller A., Roisman L., de Oliveira Dias J.R., Yehoshua Z., Schaal K.B., Feuer W. (2017). Automated quantitation of choroidal neovascularization: A comparison study between spectral-domain and swept-source OCT angiograms. Investig. Ophthalmol. Vis. Sci..

[35] Taibouni K., Chenoune Y., Miere A., Colantuono D., Souied E., Petit E. (2019). Automated quantification of choroidal neovascularization on Optical Coherence Tomography Angiography images. Comput. Biol. Med..

[36] Vaghefi E., Hill S., Kersten H.M., Squirrell D. (2020). Multimodal retinal image analysis via deep learning for the diagnosis of intermediate dry age-related macular degeneration: A feasibility study. J. Ophthalmol..

[37] Wang J., Hormel T.T., Gao L., Zang P., Guo Y., Wang X., Bailey S.T., Jia Y. (2020). Automated diagnosis and segmentation of choroidal neovascularization in OCT angiography using deep learning. Biomed. Opt. Express.

[38] Mohammad S. (2015). Textural Measurements for Retinal Image Analysis. Ph.D. Thesis.

[39] Liu L., Chen J., Fieguth P., Zhao G., Chellappa R., Pietikäinen M. (2019). From BoW to CNN: Two decades of texture representation for texture classification. Int. J. Comput. Vis..

[40] Liu L., Fieguth P., Guo Y., Wang X., Pietikäinen M. (2017). Local binary features for texture classification: Taxonomy and experimental study. Pattern Recognit..

[41] Pietikäinen M., Hadid A., Zhao G., Ahonen T. (2011). Computer Vision Using Local Binary Patterns.

[42] Ojala T., Pietikainen M., Harwood D. (1994). Performance evaluation of texture measures with classification based on Kullback discrimination of distributions. Proceedings of the 12th International Conference on Pattern Recognition.

[43] Ojala T., Pietikäinen M., Harwood D. (1996). A comparative study of texture measures with classification based on featured distributions. Pattern Recognit..

[44] Calonder M., Lepetit V., Strecha C., Fua P. (2010). Brief: Binary robust independent elementary features. Proceedings of the European Conference on Computer Vision.

[45] Calonder M., Lepetit V., Ozuysal M., Trzcinski T., Strecha C., Fua P. (2011). BRIEF: Computing a local binary descriptor very fast. IEEE Trans. Pattern Anal. Mach. Intell..

[46] Morris T., Mohammed S. (2015). Characterising glaucoma using texture. Proceedings of the 19th Conference on Medical Image Understanding and Analysis.

[47] Mohammad S., Morris D., Thacker N. (2013). Texture analysis for the segmentation of optic disc in retinal images. Proceedings of the 2013 IEEE International Conference on Systems, Man, and Cybernetics.

[48] Albarrak A., Coenen F., Zheng Y. Age-related macular degeneration identification in volumetric optical coherence tomography using decomposition and local feature extraction. Proceedings of the 17th Medical Image Understanding and Analysis Conference.

[49] Liu Y.Y., Chen M., Ishikawa H., Wollstein G., Schuman J.S., Rehg J.M. (2011). Automated macular pathology diagnosis in retinal OCT images using multi-scale spatial pyramid and local binary patterns in texture and shape encoding. Med. Image Anal..

[50] Ali M.A., Hurtut T., Faucon T., Cheriet F. (2014). Glaucoma detection based on local binary patterns in fundus photographs. Proceedings of the Medical Imaging 2014: Computer-Aided Diagnosis.

[51] Liu Y.Y., Chen M., Ishikawa H., Wollstein G., Schuman J.S., Rehg J.M. (2010). Automated macular pathology diagnosis in retinal OCT images using multi-scale spatial pyramid with local binary patterns. Proceedings of the International Conference on Medical Image Computing and Computer-Assisted Intervention.

[52] Balaskas K., Alfahaid A., Khalid H., Sergouniotis P., Pontikos N., Keane P.A. (2019). Machine Learning for the automated interpretation of Optical Coherence Tomography Angiography for Age-related Macular Degeneration. Investig. Ophthalmol. Vis. Sci..

[53] Alfahaid A., Morris T., Cootes T., Keane P.A., Khalid H., Pontikos N., Sergouniotis P., Balaskas K., Zheng Y., Williams B.M., Chen K. (2020). A Hybrid Machine Learning Approach Using LBP Descriptor and PCA for Age-Related Macular Degeneration Classification in OCTA Images. Proceedings of the Medical Image Understanding and Analysis.

[54] Alfahaid A., Morris T., Nixon M., Mahmoodi S., Zwiggelaar R. (2018). An Automated Age-Related Macular Degeneration Classification Based on Local Texture Features in Optical Coherence Tomography Angiography. Proceedings of the Medical Image Understanding and Analysis.

[55] Ahonen T., Hadid A., Pietikainen M. (2006). Face description with local binary patterns: Application to face recognition. IEEE Trans. Pattern Anal. Mach. Intell..

[56] Noble W.S. (2006). What is a support vector machine?. Nat. Biotechnol..

[57] Cunningham P., Delany S.J. (2020). k-Nearest Neighbour Classifiers. arXiv.

[58] James G., Witten D., Hastie T., Tibshirani R. (2013). An Introduction to Statistical Learning.

[59] Pedregosa F., Varoquaux G., Gramfort A., Michel V., Thirion B., Grisel O., Blondel M., Prettenhofer P., Weiss R., Dubourg V. (2011). Scikit-learn: Machine Learning in Python. J. Mach. Learn. Res..

[60] Itseez (2015). Open Source Computer Vision Library. https://github.com/itseez/opencv.

[61] Liu Y., Cheng D., Zhang D., Xu S., Han J. (2024). Capsule networks with residual pose routing. IEEE Trans. Neural Netw. Learn. Syst..

